# High-throughput ultrastructural analysis of macular telangiectasia type 2

**DOI:** 10.3389/fopht.2024.1428777

**Published:** 2024-07-30

**Authors:** Charles L. Zucker, Paul S. Bernstein, Richard L. Schalek, Jeff W. Lichtman, John E. Dowling

**Affiliations:** ^1^ Department of Molecular and Cellular Biology, Harvard University, Cambridge, MA, United States; ^2^ Department of Ophthalmology and Visual Sciences, Moran Eye Center, University of Utah School of Medicine, Salt Lake City, UT, United States; ^3^ Center for Brain Science, Harvard University, Cambridge, MA, United States

**Keywords:** macular telangiectasia type 2, MacTel, serine, Müller cell, microglia, mitochondria, macular degeneration, electron microscopy

## Abstract

**Introduction:**

Macular Telangiectasia type 2 (MacTel), is an uncommon form of late-onset, slowly-progressive macular degeneration. Associated with regional Müller glial cell loss in the retina and the amino acid serine synthesized by Müller cells, the disease is functionally confined to a central retinal region – the MacTel zone.

**Methods:**

We have used high-throughput multi-resolution electron microscopy techniques, optimized for disease analysis, to study the retinas from two women, mother and daughter, aged 79 and 48 years respectively, suffering from MacTel.

**Results:**

In both eyes, the principal observations made were changes specific to mitochondrial structure both outside and within the MacTel zone in all retinal cell types, with the exception of those in the retinal pigment epithelium (RPE). The lesion areas, which are a hallmark of MacTel, extend from Bruch’s membrane and the choriocapillaris, through all depths of the retina, and include cells from the RPE, retinal vascular elements, and extensive hypertrophic basement membrane material. Where the Müller glial cells are lost, we have identified a significant population of microglial cells, exclusively within the Henle fiber layer, which appear to ensheathe the Henle fibers, similar to that seen normally by Müller cells.

**Discussion:**

Since Müller cells synthesize retinal serine, whereas retinal neurons do not, we propose that serine deficiency, required for normal mitochondrial function, may relate to mitochondrial changes that underlie the development of MacTel. With mitochondrial changes occurring retina-wide, the question remains as to why the Müller cells are uniquely susceptible within the MacTel zone.

## Introduction

If we are to understand retinal diseases and to design rational therapies for their cure, it is essential that we understand their etiology. One thing we need to know are the initial changes that occur in any retinal disease, and this requires high resolution microscopy, namely electron microscopy (EM) which can visualize tissue organization, cellular structure, and even subcellular organelles. Diabetic retinopathy is one example. Although it is usually diagnosed because of retinal vascular changes, more recent evidence suggests it may begin in retinal neurons and Müller glia (1–3). Similarly, there are significant gaps in our understanding of the root causes of glaucoma and AMD. High-throughput ultrastructural analysis might reveal the cells, whether vascular, neuronal or glial, that first show changes and their nature.

The ability to “drill-down” to clues about etiology using ultrastructural imaging, has been limited historically by the narrow field of view inherent in very high-resolution techniques. Recent advances in large-scale serial-section EM, connectomics, have vastly broadened the field of view, allowing for the comprehensive reconstruction of relatively large pieces of brain tissue, including the retina, in three dimensions (4–6). Using animal models of retinal diseases, such methods, referred to as retinal pathoconnectomics, are being applied to investigate the remodeling and rewiring of the retina that occurs as a consequence of retinal disease (7, 8).

We have used a connectomics approach, optimized for investigating disease processes that occur over geographically large areas of human donor retinas, to investigate the etiology of a rare form of age-related maculopathy, macular telangiectasia type 2 (MacTel). MacTel is a slowly-progressive macular degeneration associated with Müller glial cell loss in the retina and likely the amino acid serine, which is synthesized exclusively by Müller cells in the neural retina (9–13). The clinically significant manifestation of the disease is restricted mainly to a central retinal region referred to as the MacTel zone, which is approximately 3mm (horizontal) by 2.5 mm (vertical), inclusive of the fovea and much of the macula. Although some visual changes can occur in the perifovea outside the MacTel zone, good vision is typically preserved there, allowing the analysis, in a single eye, of a region with significant visual loss and regions functionally relatively normal. Discrete pigmented lesions or plaques in the MacTel zone cause scotomas (blind spots) and dramatic losses of visual sensitivity in the affected areas (14–16). MacTel patients complain of metamorphopsia – straight lines appear wavy or bent. In advanced cases, retinal vessels, at times with acute angles or ending abruptly, encroach into the avascular zone around the lesion area, hence the name – macular telangiectasia, however it is thought that these vascular abnormalities are secondary to photoreceptor loss. Local cavitations, associated with distortions around the lesions, also occur (17). MacTel patients typically show a loss of the yellow macular pigment resulting in blue light reflection abnormalities, along with the presence of “crystals” in the inner retina of unknown composition (18). This loss of macular pigment coincides with the region of Müller cell loss, supporting the idea that these pigments reside in Müller cells (9, 19–23).

Using our multi-resolution targeted high-throughput connectomics approach, we have investigated the cellular and subcellular changes that have occurred in the retinas from a mother and daughter, aged 79 and 48, both suffering from MacTel (provided to us by Paul Bernstein). As we reported previously, initial analysis of the 48 year old donor retina showed structural changes in the mitochondria in horizontal and photoreceptor cells, both within and several millimeters outside the MacTel zone. We also described a transition zone that represents an abrupt boundary defining the limit of the MacTel zone where macular pigment and Müller cells are lost (22, 24). The MacTel transition zone likely represents a region where the disease process and the cellular attempts at mitigation is prominently manifest, with active neuronal and glial degeneration.

In the macula region of the primate retina, the photoreceptor axons (Henle fibers) and synaptic terminals are normally isolated from each other by ensheathing Müller glial cell processes (25). We found a striking demarcation along this boundary marked by profound loss of Müller cell ensheathment of Henle fibers that is well maintained on the other side of the border, barely 100 μm away. The Henle fiber layer is normally devoid of cell bodies, with the nearest cell bodies being located in the overlying outer nuclear layer.

In the present report, we provide more in-depth analysis of our 48 year old donor retina and extend our findings to include the retina from our 79 year old donor. This analysis of both donor retinas now extends from the choriocapillaris and retinal pigment epithelium (RPE), through the full extent of the lesion areas (with their associated vascular connections), the prevalence of microglial cells within the Henle fiber layer, as well as the structural integrity through the retinal layers down through the inner limiting membrane. Our observations, which show consistencies between both donors, provide clues about the causes and cellular responses to degeneration of the retina in MacTel. This analysis also highlights the utility of our multi-resolution high-throughput connectomics approach in gaining an understanding of the cellular and subcellular basis for a range of degenerative diseases affecting vision; a level of mechanistic understanding not possible with the most powerful forms of clinical imaging.

## Materials and methods

### Procurement of human donor eye

The MacTel eyes used in the present study were provided by Dr. Paul Bernstein at the University of Utah. The donors were a 48 year old female who died of metastatic uterine leiomyosarcoma approximately 4 years after being diagnosed with MacTel and her 79 year old mother who died approximately 16 years after being diagnosed with MacTel. We have not been provided with any identifiers associated with the donors other than cause of death of the daughter and information specific to the retinal disorder. All such information remains with Dr. Bernstein in accordance with all University of Utah privacy of research participants and confidentiality of data protocols.

### Tissue preservation and initial processing

The eyes from the 48 year old donor were collected 94 minutes after death, with the left eye (OS) being fully processed and placed into one-half strength Karnovsky’s fixative in cacodylate buffer after 2 hours, 32 minutes of death. The right eye (OD) was fixed for light microscopical studies, with DNA, sera, and plasma also collected for studies performed in other laboratories. Initial photography and trimming into a Maltese cross occurred prior to our obtaining the tissue. Once received, the tissue, still attached to the sclera, was transferred into fresh half-strength Karnovsky’s and stored at 4°C prior to further processing. Similar parameters also applied to the eyes of the 79 year old donor, with the left eye (OS) being fixed for electron microscopy. Total storage time in fixative for each eye was approximately 6 years prior to further processing.

### Processing for serial section electron microscopy

Except where noted, tissue handling and processing was identical for the retinas from both donors. Following multiple rinses in cold 0.1M cacodylate buffer w/0.15 mM CaCl_2_, the sclera was removed from the retina with attached pigment epithelium. The retinas were dissected down as follows: Forty-eight YO to an area ~3 mm nasal/temporal x 2.5 mm inferior/superior inclusive of the fovea, the MacTel zone, and surrounding areas; Seventy-nine YO to an area ~6 mm nasal/temporal x 4 mm inferior/superior inclusive of the fovea, the MacTel zone, and surrounding areas. Since further processing of such large pieces of retina would typically induce significant curling, we designed a processing chamber within which the retina is held flat while allowing free exchange of fixatives and other reagents. Within the chamber, the retina is held by nickel micromesh sheets with an open area of 78% (Precision eForming, Cortland, NY) separated by Teflon spacers equal to the thickness of the retina. The chamber components are also nonreactive to the reducing agents and other reagents required for the reduced osmium impregnation protocols used to render the tissue electron dense and conductive suitable for scanning EM imaging. The tissue was stained using a modified ROTO procedure (4). In brief, the processing chamber with mounted tissue was immersed in reduced osmium tetroxide (1.5% potassium ferrocyanide/2%OsO_4_ in cacodylate buffer) on ice for 1 hour. Following washout with distilled water, tissue was incubated for 20 minutes in 1% room temperature thiocarbohydrazide, water rinsed, then incubated for 30 minutes in 2% aqueous OsO_4_. The tissue was then aqueous washed and en-bloc stained overnight in 1% aqueous uranyl acetate at 4°C. Following thorough aqueous washout and a 30 minute incubation in Walton’s lead nitrate at 60°C (0.066g lead nitrate in 10ml 0.03M aspartic acid solution pH 5.5), the tissue was dehydrated to absolute ethanol before removal from the incubation chamber. Tissue was then exchanged into propylene oxide then infiltrated with ascending concentrations of epon 812 over the course of 24 hours. While in 100% liquid resin, pieces were cut off for mounting for radial sections (nasal to the fovea but including part of the MacTel zone of the 48YO retina, and just temporal to the presumed border of the MacTel zone for the 79 YO retina) with the remaining tissue being flat embedded in epon between aclar sheets and remounted onto a blank epon block for trimming and sectioning.

### Serial sectioning, imaging, and analysis

The blocks were initially trimmed by collecting 1 μm sections, stained with a methylene blue/toluidine blue solution and observed light microscopically to determine depth and orientation of cutting prior to ultrathin sectioning. Approximately 10,000 serial sections at 30 nm (48YO) or 60 nm (79 YO) were collected using an automated tape-collecting ultramicrotome (ATUM) (5, 6, 26). Strips of tape with the serial sections were mounted onto silicon wafers with double-sided carbon tape, plasma treated and post-stained with uranyl acetate and lead citrate prior to imaging. For the 48 YO retina, the first and middle section from each wafer (each wafer contains close to 200 sections) was imaged in its entirety at an XY resolution of 70 nm in a Zeiss Sigma field-emitting scanning electron microscope to create an overview image stack to aid in the selection of regions of interest (ROIs). Selected regions in X, Y, and Z position were then reimaged at 4 nm X/Y resolution using either the Zeiss Sigma or Zeiss 61-beam MultiSEM depending on the Z extent and size of each ROI. Images were stitched and aligned prior to observation and analysis using the Volume Annotation and Segmentation Tool (VAST; 27). Mapping of microglial cells near the fovea and along the MacTel zone border was performed by segmenting the full extent of each microglial cell nucleus, as identified by their distinct morphological characteristics, as well as the properties of the surrounding cytoplasm. For the 79 YO retina, a single section from the middle of every approximately 5^th^ wafer was imaged in its entirety at an XY resolution of 60 nm in a Zeiss Sigma field emitting scanning electron microscope. Selected regions in X, Y, and Z position from the radial block were then reimaged at 4 nm X/Y resolution using the Zeiss Sigma EM.

### Data availability

Each volume dataset, after image stitching, alignment and generation of the volume will be housed on the Harvard server for free-access and unfettered use by other investigators after completion of the datasets. Once a volume is placed on the Harvard site it will be available as an open mineable database. All software and analysis tools developed by the Lichtman lab will be made available to the scientific community upon request.

## Results

### Clinical, light-microscopic, and µCT X-ray imaging

**Figure 1:**
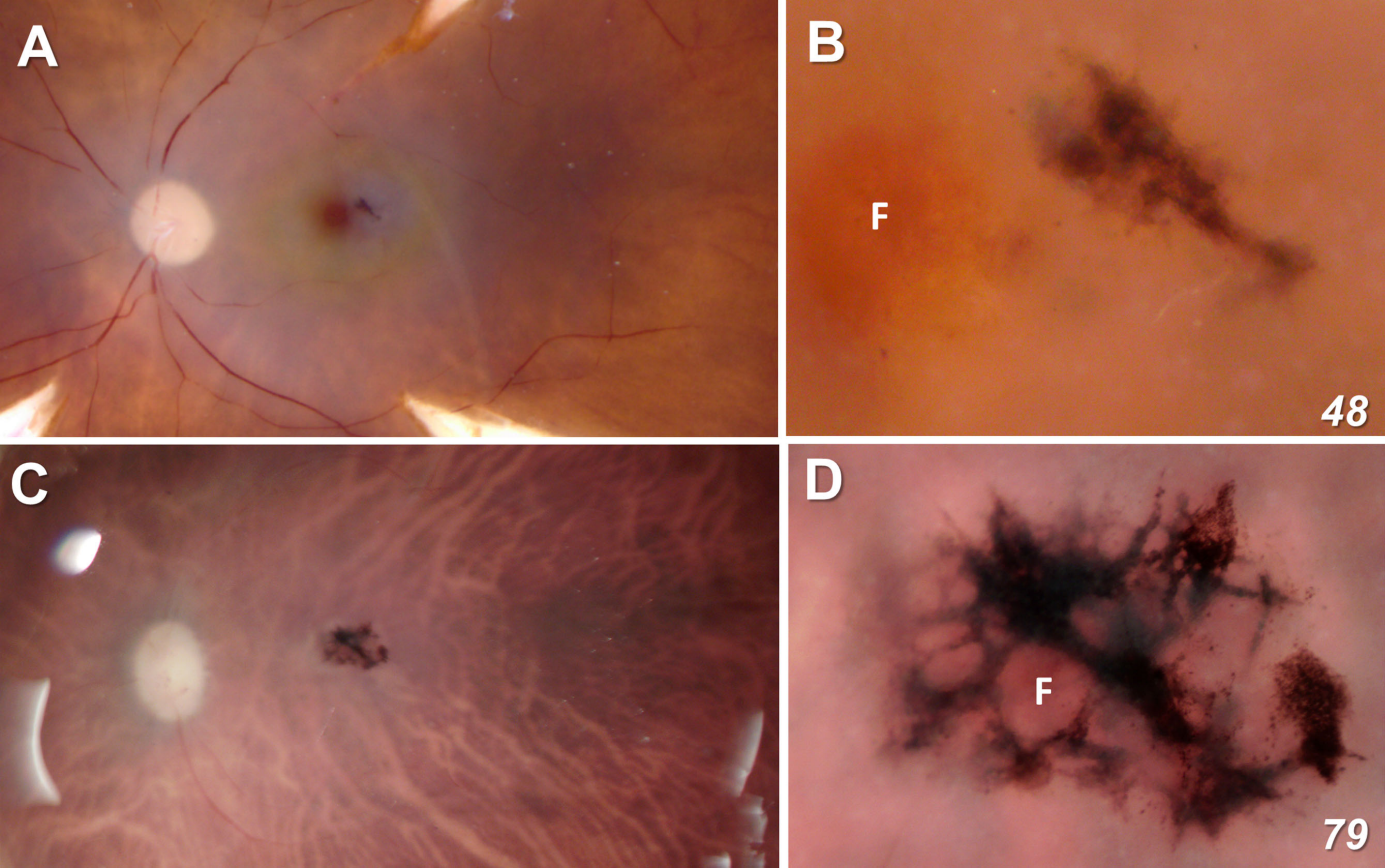
Fundus view of our two MacTel donor eyes (**A**, **B** = 48 year-old; **C**, **D** = 79 year-old). Figures **(A, C)** are shown at equal scale. The lesion in the 48-year-old retina is ~200 μm x 400 μm and completely outside the foveal center F. In the 79-year-old donor, the lesion is ~ 1.5 mm x 1 mm and surrounds completely the fovea as defined by the center point of the rod-free zone.

At the ophthalmoscopic level, a defining feature of MacTel is a discrete pigmented lesion, typically on the temporal side of the fovea, near the center of the MacTel zone where macular pigment is also lost. These features are seen clearly in our 48-year-old donor eye (48YO), with an approximately 200 μm x 400 μm lesion temporal to the fovea (**Figures 1A, B**). Although we were not able to visualize the macular pigment in the 79-year-old donor eye (79YO; perhaps due to the age of the donor, or to histological factors), the lesion is much more extensive in her eye, at approximately 1.5 mm x 1 mm, with it surrounding the fovea completely (**Figures 1C, D**). **Figures 1A, C** are shown at equal scale.

**Figure 2:**
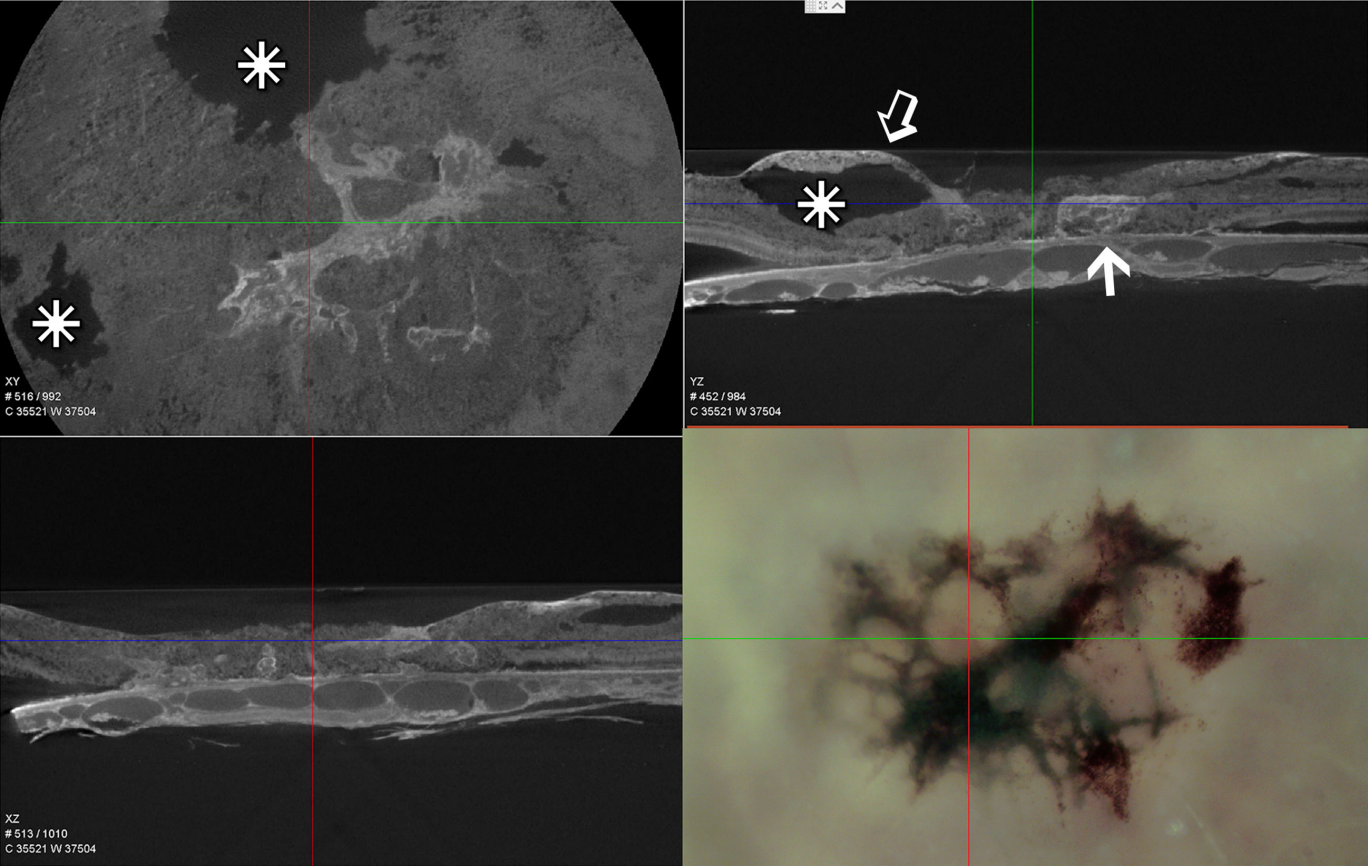
Single slice of low resolution μCT – XY, YZ, and XZ projections of the lesion area of the 79-year-old donor eye and a light micrograph of the same area prior to processing for EM. Similarly scaled, the cross hairs in all four images are centered on the foveal center. The osmiophilic lesion is seen to be continuous with Bruch’s membrane and the underlying choriocapillaris (arrow), while extending above (open arrow) and retinal cavitations (stars).

Based on interference of X-ray transmission, our tissue preparation techniques that use enhanced heavy metal staining for our electron microscopic imaging are also optimal for μCT X-ray imaging. The lesions themselves are highly pigmented and osmiophilic due to numerous inclusion bodies of various types, as well as the migration of cells from the overlying RPE (**Figure 2**). Although our μCT instrument was not obtained until after our 48YO donor retina was already sectioned, X-ray imaging of our 79YO donor retinal reveals several large retinal cavitations in the XY and YZ projections (**Figure 2**). Such cavitations are also described clinically. At the depth of the RPE, a close association with part of the lesion is indicated just temporal to the fovea (**Figure 2**). This plane of section corresponds to that shown ultrastructurally in **Figure 9A** which corresponds to the origination of the MacTel lesion above and below Bruch’s membrane.

**Figure 3:**
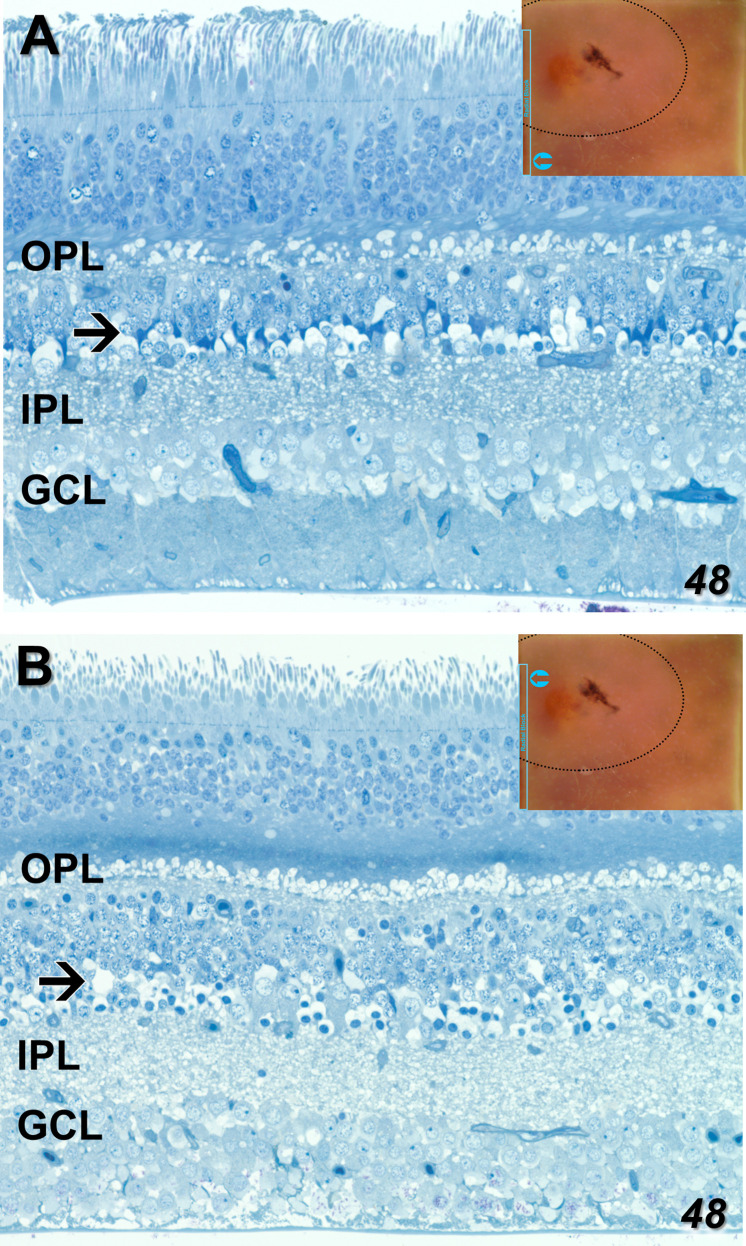
One-micron sections for light microscopy were taken immediately prior to EM sectioning of our radially mounted blocks. Parts of a single section spanning from outside to inside the MacTel zone from our 48-year-old retina are shown (An analogous section from our 79-year-old retina is shown in **Supplementary Figure S1**). Outside the MacTel zone **(A)**, some swelling in the outer plexiform layer (OPL) is seen surrounding many of the photoreceptor terminals. In the proximal inner nuclear layer (arrow), darkly staining Müller cell cytoplasm (typical of Müller cells), is seen above many of the amacrine cell bodies. The ganglion cell layer (GCL) and the underlying nerve fiber bundles appear healthy. Inside the MacTel zone **(B)**, swellings above the photoreceptor terminals, likely of Müller cell origin, are seen consistently along the OPL. Just distal to the OPL, a dark band in the Henle fiber layer may represent the microglial cells we observe ultrastructurally (see **Figure 11**). Unlike outside the MacTel zone, darkly staining Müller cell cytoplasm is missing (arrow), and there is greater disorder of the nerve fiber bundles. Insets show the location of the images taken from an individual radial section.

Just prior to acquiring radial sections for EM on the nasal side of the fovea from the 48YO donor, several 1 μm sections were taken that spanned from inside the MacTel zone to beyond its border. Light microscopy hints at many MacTel features we describe ultrastructurally. Outside the MacTel zone, the darkly staining cytoplasm characteristic of Müller cell bodies is seen clearly in the inner nuclear layer (**Figure 3A**). The inner limiting membrane, composed of the Müller cell end-feet, along with the ganglion cell axon bundles also maintain good integrity. Similar features are seen outside the MacTel zone of the 79YO donor, with prominent dark Müller cell cytoplasm (**Supplementary Figure S1**). In the outer plexiform layer, patchy swellings just distal (above) the photoreceptor synaptic terminals are seen (**Figure 3A**). These swollen profiles likely represent Müller cell processes that normally ensheathe the terminals (25). Inside the MacTel zone (**Figure 3B**), darkly stained Müller cell cytoplasm is no longer visible in the inner nuclear layer, while the ganglion cell axons are less tightly bundled. In the outer plexiform layer, clear profiles distal to the photoreceptor terminals are seen consistently. In the Henle fiber layer, which is thicker than outside the MacTel zone due to its proximity to the foveal center, a more darkly staining band was noted in every section we cut for light microscopy. Consistent with our ultrastructural findings, this band may represent the prevalence of microglial cells that we find interspersed with the Henle fibers exclusively within the MacTel zone (see **Figure 11**).

### EM – Neural retina

Despite a significant age difference between the two donors, just outside the MacTel zone we observe a remarkable consistency in the cellular and mitochondrial changes that manifest in both. Consistent with what we reported previously for the 48YO (22), mitochondrial changes outside the MacTel zone in the 79YO include a reduction in cristae, swelling with an increase in matrix area, and inclusion of electron-dense material, are seen in neurons throughout the depth of the retina from the ganglion cells up through the photoreceptors.

**Figure 4:**
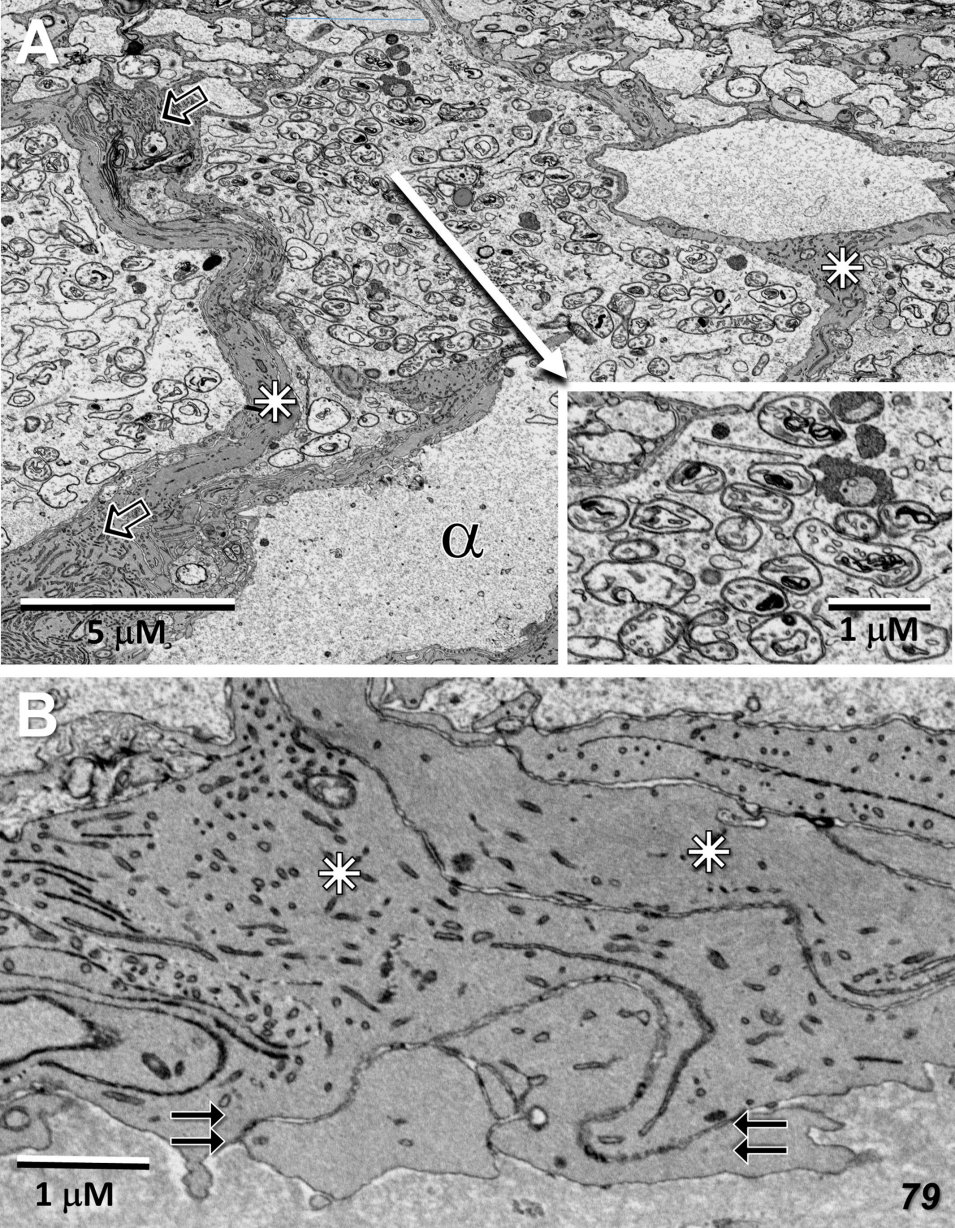
Radial sections of the ganglion cell layer and inner limiting membrane temporal to the 79-year-old MacTel zone. **(A)** Just beyond the plane of the nucleus, a ganglion cell body is densely filled with mitochondria and other organelles which, as is normal, are not present in the connecting axon initial segment (alpha). Nearly all of the mitochondria show signs of pathology, such as condensed cristae (inset). Müller cell processes, filled with vimentin filaments (open arrows) surround and stream around the ganglion cells (stars). **(B)** The end-feet from multiple Müller cell processes (stars) juxtapose (double-arrows), forming an inner limiting membrane of normal appearance.

**Figure 5:**
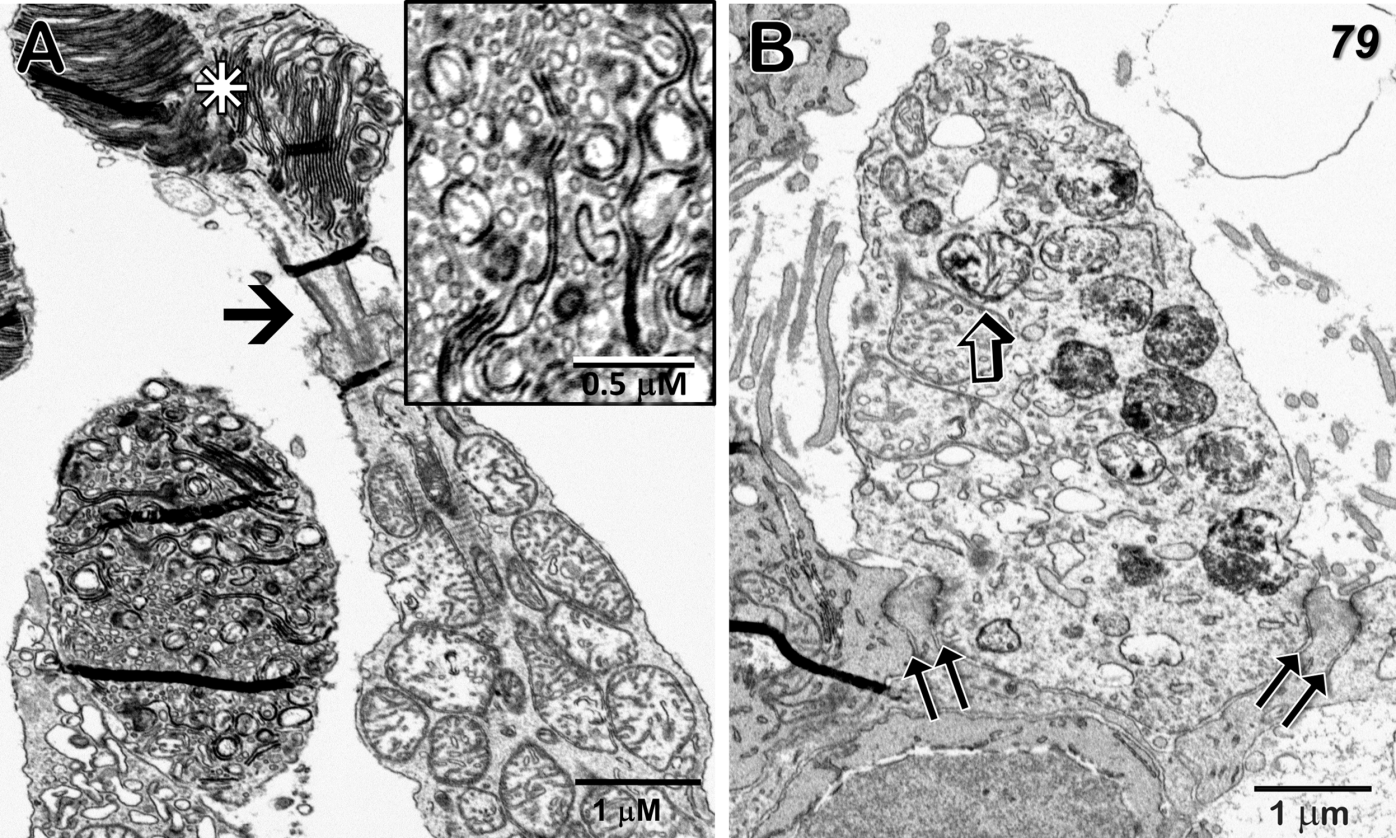
Cone outer and inner segments, and outer limiting membrane just temporal to the 79-year-old MacTel zone. **(A)** The normally highly-ordered cone outer segment discs show varying degrees of pathology; ranging from clusters of aligned discs (star) above a connecting cilium (arrow), to those exhibiting highly disordered disc membranes with extensive fragmentation and vesicularization (inset). Along with discs connected by membranous loops, little association with the outer plasma membrane is seen. **(B)** A portion of a cone inner segment ellipsoid reveals many mitochondria with isolated electron-dense cristae as well as those with more uniform dense material undergoing degradation. Müller cell processes, interspersed with photoreceptor inner segments and tight-junctions, constitute an intact outer limiting membrane (double-arrows). Some small artifactual folds that appear dense, occur most often in areas of varied density, such as where tissue is adjacent to bare epon in the block, are also visible.

Ganglion cells show a typical enrichment in mitochondria and other organelles in their cell bodies with a sharp drop-off of these organelles in their axon initial segments. Müller cell processes, interestingly are also well maintained, with a significant complement of vimentin filaments, as they surround the ganglion cells and axons on their way toward the inner limiting membrane (**Figure 4A**). Despite this generalized cellular integrity, mitochondria show an admixture of pathological features, ranging from highly condensed to those with significant areas of empty matrix devoid of cristae (**Figure 4A** inset). The inner limiting membrane, consisting of the end-feet of Müller cells, also appears normal in this region just outside the MacTel zone (**Figure 4B**), which together with the integrity of the outer limiting membrane in this region (**Figure 5B**), suggests that the Müller cells are functionally intact, and thus not correlated directly, with the mitochondrial changes we observe throughout both of our donor retinas.

**Figure 6:**
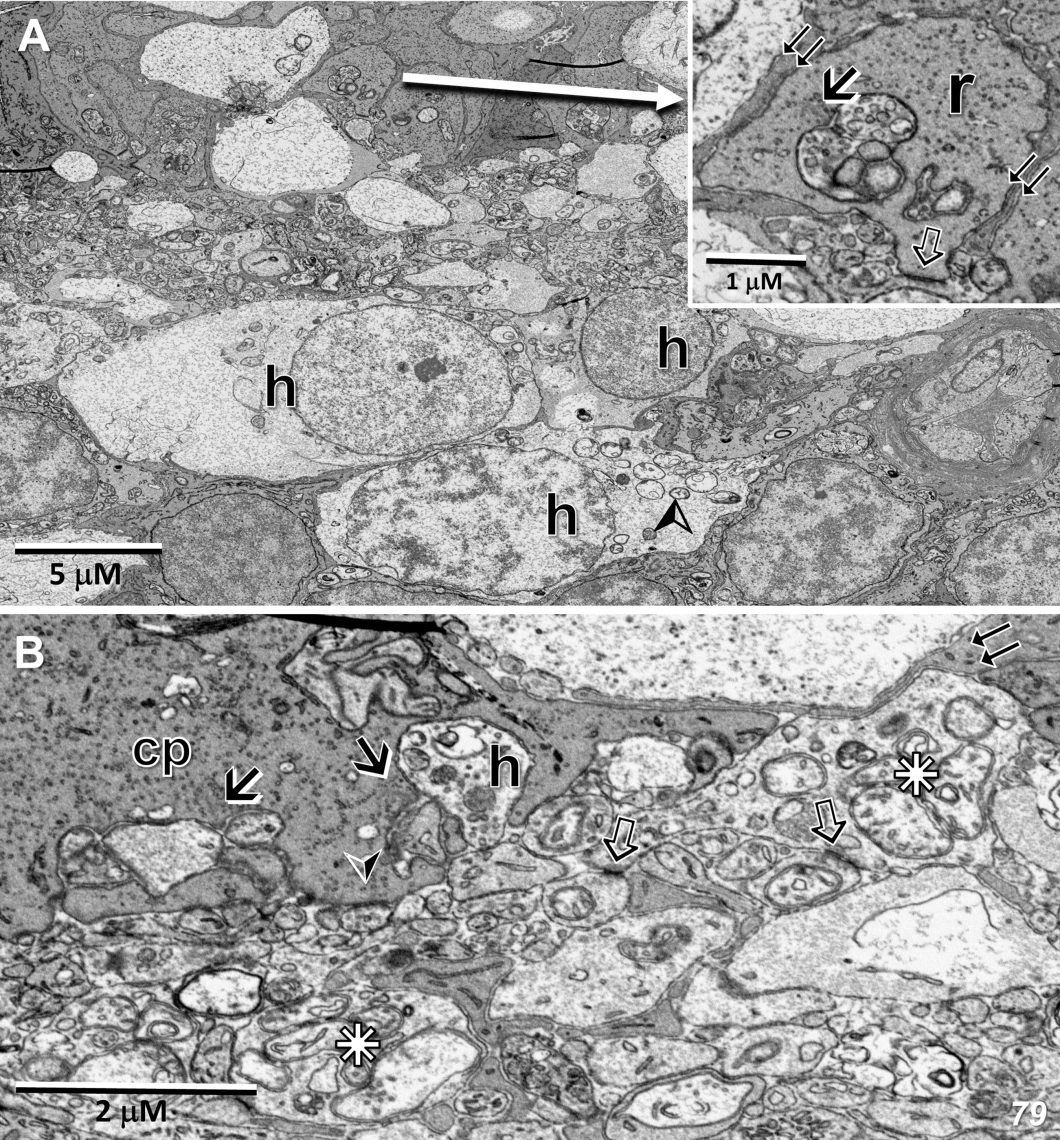
Radial sections of the outer plexiform layer temporal to the 79-year-old MacTel zone. **(A)** In this region, a rod spherule (r) is ensheathed by Müller cell processes (inset – double-arrows), while showing a synaptic ribbon (arrow) with its associated postsynaptic horizontal and bipolar cell processes, as well as a putative gap junction likely with a telodendrial process of an adjacent rod (open arrow). Several horizontal cell bodies (h), the upper two of which are likely coupled to each other, often contain swollen mitochondria (arrowhead). **(B)** A cone pedicle (cp) contains multiple synaptic ribbons (arrows) with their associated postsynaptic processes, as well as a likely basal junction (arrowhead), demonstrating a preservation of functional connectivity. Horizontal cell processes (stars), shows multiple swollen mitochondria (compare to **Figure 3B** in 22), along with several sites of likely coupling with adjacent horizontal cell processes (open arrows). Horizontal cell processes lateral to synaptic ribbons may also show swelling and mitochondrial pathology (h). Müller cell ensheathment is also maintained (double-arrows).

The mitochondria in the photoreceptor inner segments (ellipsoid region) are often filled with dense cristae, although some adjacent mitochondria are more normal in appearance (**Figure 5**). Photoreceptor outer segments are highly variable, with some showing multiple clusters of well-aligned discs, while the discs in others are highly fragmented with little organized stacking (**Figure 5A**). At the base of the inner segments, tight-junctions between the photoreceptors and Müller cell processes, that constitute the outer limiting membrane, also maintain their integrity (**Figure 5B**). Similar to what we reported for our 48YO donor (see Figure 7 in Zucker et al., 2020; 22), an abrupt change in the structure of the outer limiting membrane occurs at the border of the MacTel zone; outside the MacTel zone, the proximal rod and cone inner-segments are separated from each other by a thin layer of Müller cell cytoplasm whereas inside the MacTel zone, the outer limiting membrane is nearly devoid of Müller cell cytoplasm leaving the rod and cone cell membranes in direct juxtaposition. This loss of Müller cell contribution occurs over a distance of less than 150 μm (**Supplementary Figure S2**). Throughout the retina outside the MacTel zone, Müller cell ensheathment of the rod spherules and cone pedicles is maintained, as are the morphological correlates of functional circuitry (**Figure 6**).

Although reduced in complexity, synaptic ribbon complexes, with their associated horizontal and bipolar cell postsynaptic partners, are still readily observed outside the MacTel zone (**Figure 6A** inset, **6B**). Basal junctions are also maintained (**Figure 6B**). Despite especially prominent mitochondrial changes in the cell bodies and processes of horizontal cells, sites of likely gap-junctional coupling between adjacent horizontal cell processes are often observed (**Figure 6B**). In bipolar cell bodies and their synaptic terminals in the inner plexiform layer, the mitochondria appear misshapen, with few, but highly electron-dense cristae. At bipolar terminal synaptic ribbons, postsynaptic processes belonging to amacrine and ganglion cells also exhibit swollen mitochondria (**Supplementary Figure S3**).

### EM – RPE

**Figure 7:**
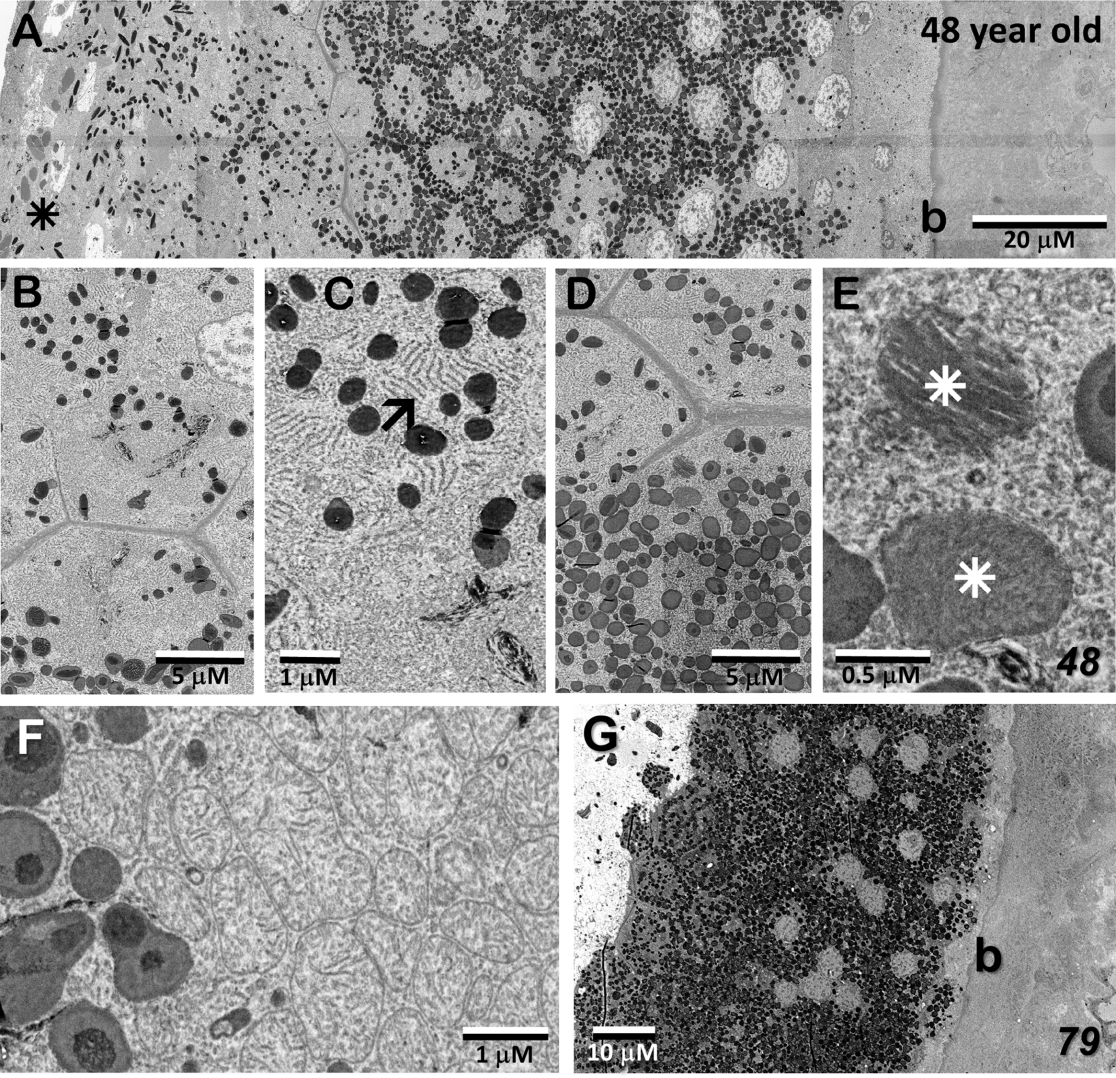
Retinal pigment epithelium in the MacTel eye. **(A–F)** from the 48 year-old donor; **(G)**, the 79-year-old donor **(A)** The full extent of the RPE is shown inside the MacTel zone. The tips of photoreceptors are on the left (star), Bruch’s membrane (b) leads to the choroid on the far right. **(B, C)** Higher magnification of RPE cells showing melanosomes and endoplasmic reticulum (arrow) inside the MacTel zone. No phagosomes containing outer segment disc material were noted. Outside the MacTel zone **(D, E)**, phagosomes were found to be present (stars), but rare. **(F)** Mitochondria in RPE cells appear normal. Like in the 48 year old, beyond the immediate lesion area, the RPE and Bruch’s membrane appear relatively normal within the MacTel zone **(G)**.

In normal retinas, a hallmark of RPE function and structure is the phagocytosis of the tips of photoreceptor outer segments. In agreement with the results of Powner et al., 2018 (28), we find few phagosomes in the RPE both inside and outside of the MacTel zone in the 48YO retina (**Figures 7A–E**). Despite this dearth of phagocytic activity, and our previous results showing mitochondrial changes in the outer retina within and beyond the MacTel zone, we observe that the mitochondria within the RPE are ultrastructurally normal in appearance (**Figure 7F**). Other cellular features of the RPE and the underlying Bruch’s membrane, from both donor eyes, also appear to maintain structural integrity (**Figures 7A–G**).

### EM – MacTel lesion; origin, extent, and vascular interaction

**Figure 8:**
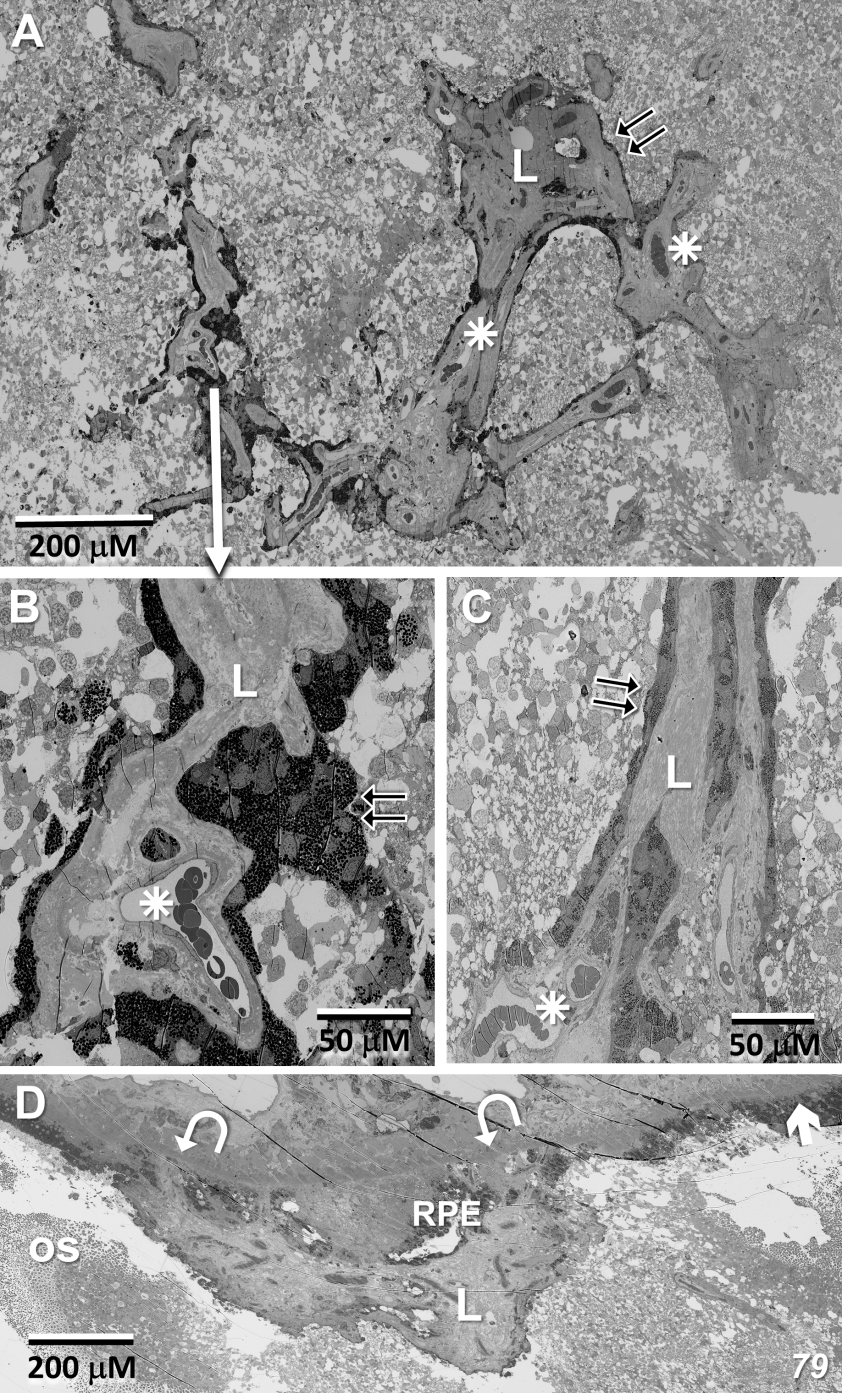
Lesion in the 79-year-old donor retina. **(A)** Near the depth of the inner nuclear layer, RPE cells migrate and surround (double-arrows) the internal vascular elements (stars), constituting the primary lesion which also contains thickened endothelial basement membranes (L). **(B, C)** Higher magnification of the lesion at the depth of the inner nuclear layer, and near the ganglion cell layer, respectively. **(D)** Marking the initiation of the lesion, one sees a breakdown in the integrity of Bruch’s membrane (arrow/curved arrows), an infiltration of vascular elements, including endothelial cells from the choriocapillaris along with migration of pigment epithelial cells through the photoreceptor layer (os – outer segments).

**Figure 9:**
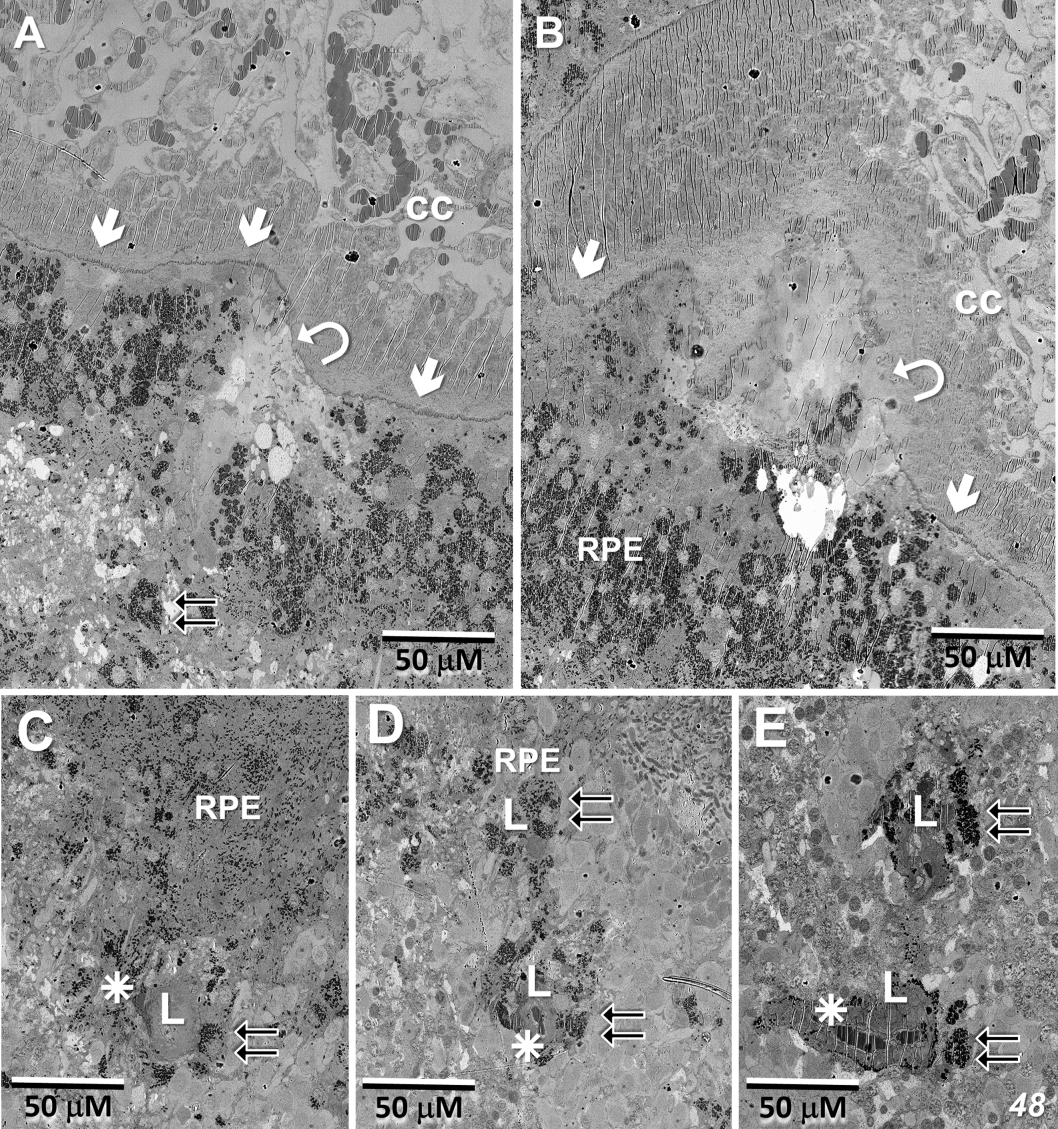
Lesion initiation and extension in the 48-year-old donor retina. **(A, B)** Similar to what we observe in the 79YO retina, a pair of sections, roughly 6 μM apart in depth, show a breakdown in the integrity of Bruch’s membrane (arrows/curved arrows) with an infiltration of vascular elements, including endothelial cells from the choriocapillaris (cc), along with migration of pigment epithelial cells through the photoreceptor layers (double-arrows). **(C–E)** Progressing proximally into the retina, RPE cells continue to migrate (double-arrows) along vascular elements (stars), constituting the primary lesion (L).

**Figure 10:**
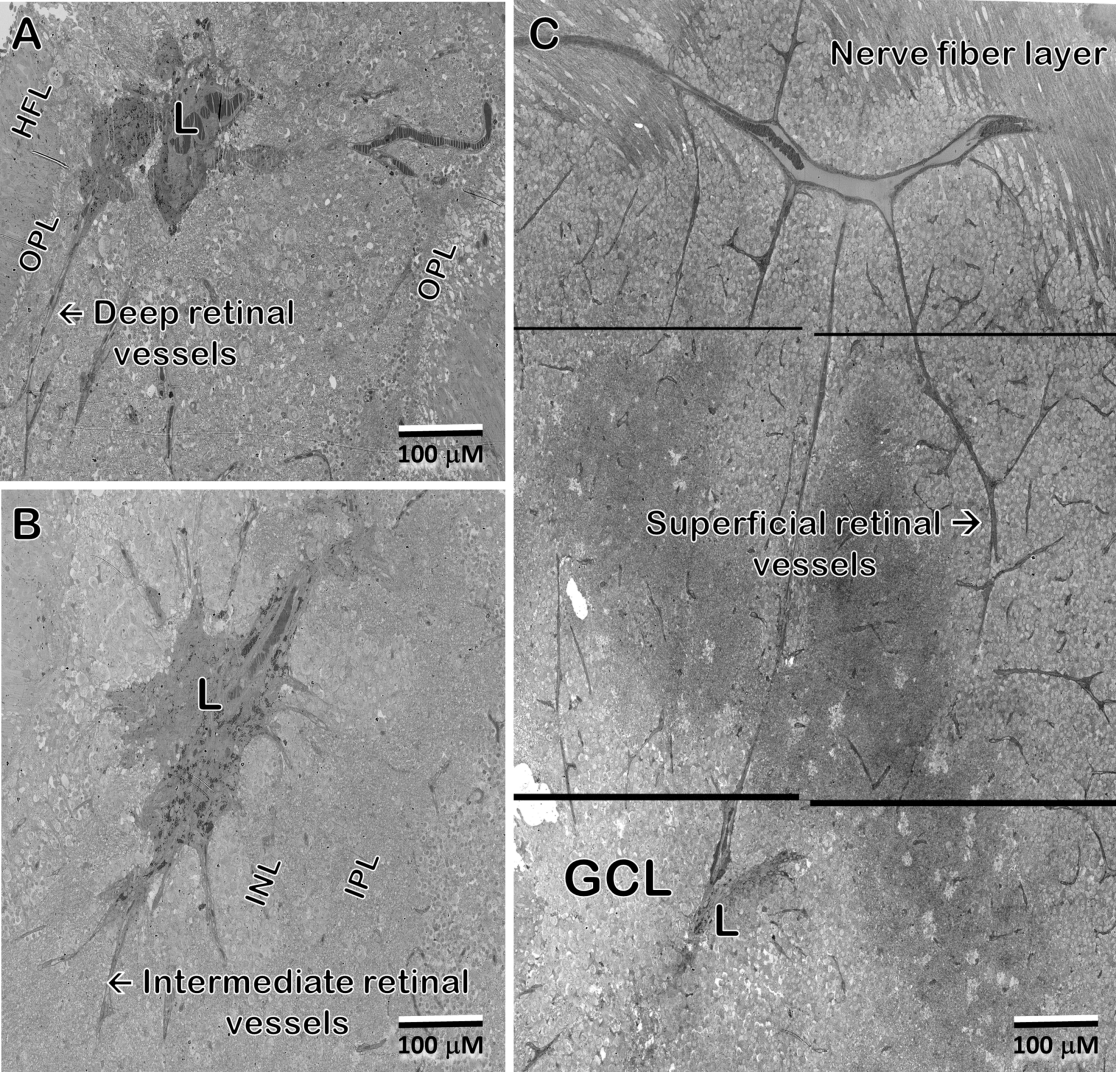
In the 48 year-old donor retina, sections at the Henle fiber/outer plexiform layer level **(A)**; inner nuclear/plexiform layer level **(B)**; and the ganglion cell/nerve fiber level **(C)** show infiltration from the deep, intermediate, and superficial retinal vascular plexes, respectively, into the lesion and integration with the vessels inside the lesion. Note that the lesion extends through the depth of the retina into the ganglion cell layer. Pigment granules are also seen at all depths.

**Figures 8A–C** show portions of the lesion in the 79YO retina. Throughout its extent, pigment epithelial cells are found within the lesion, and especially surrounding its peripheral borders. Between the pigment epithelial cells and the capillary lumens, the outer basement membrane is highly hypertrophic, similar to that described with age and in diabetic retinas (29–31). As suggested by the μCT images from our 79YO donor (**Figure 2**), in both donor retinas, the lesions begin as a focal breakdown of Bruch’s membrane on the temporal side of the fovea, allowing vascular elements from the choriocapillaris, including extensive basement membrane material from capillary endothelial cells, to infiltrate into the retinal layers, along with cells from the RPE (**Figures 8D**, **9A, B**). Although both lesions differ significantly in their XY extent, both extend from the level of Bruch’s membrane, extending through the depth of the retina to the ganglion cell layer (**Figures 8**, **9**; see also **Figure 10**). Vascularization of the lesions is also maintained throughout the retina with contributions from the deep, intermediate, and superficial retinal vessels as one progresses through the depth of the retina (**Figure 10**).

### EM – Microglia and the MacTel zone border

**Figure 11:**
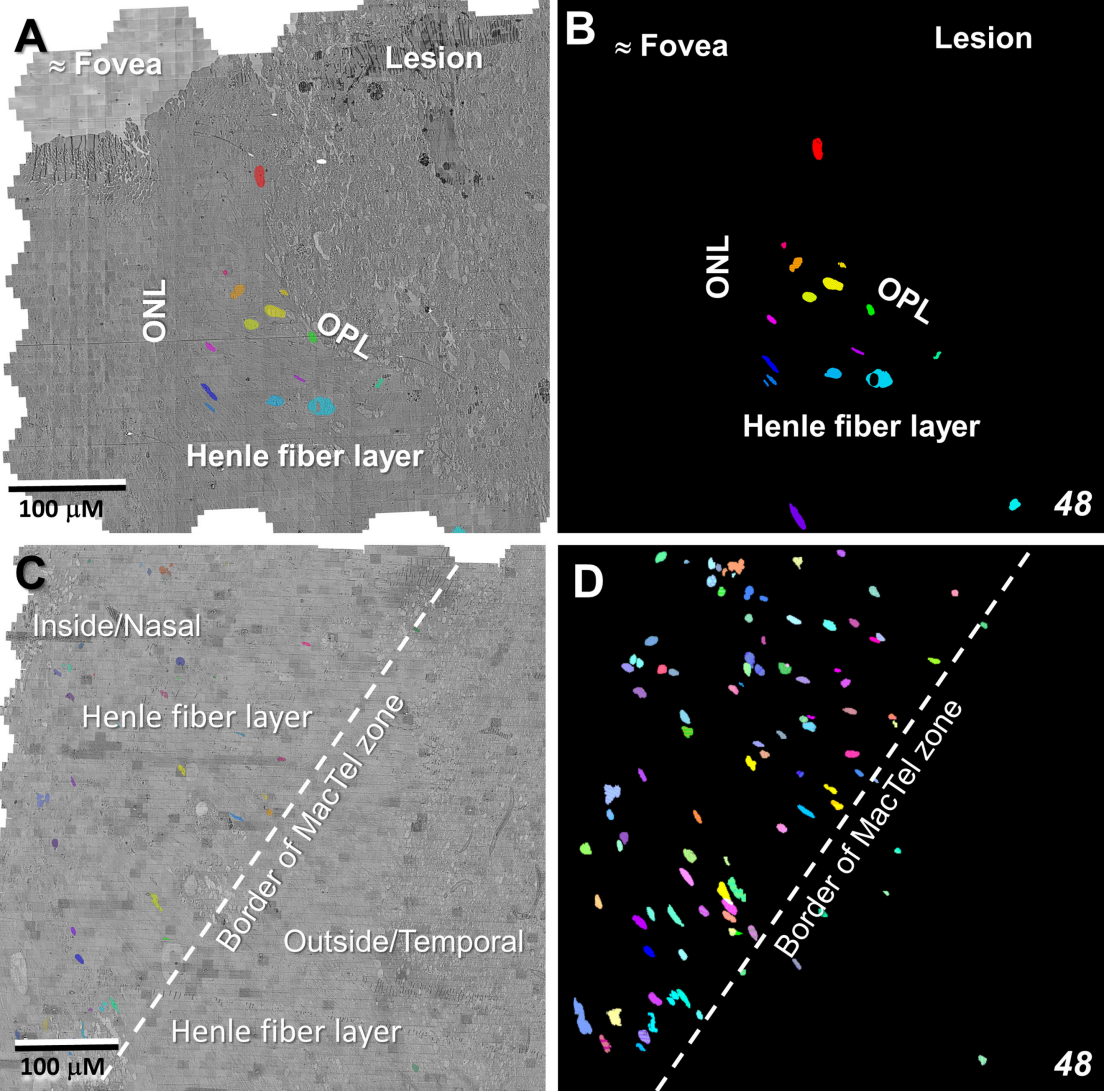
Microglial cells populate densely the Henle fiber layer inside the MacTel zone. Segmentation of microglial cell nuclei (as proxy for the location of each individual microglial cell), shows that they are limited to the Henle fiber layer from near the foveal center **(A, B)** out to the transition zone border **(C, D)**. Beyond the border and in the retinal layers immediately above and below the Henle fiber layer, few microglial cells are found. This microglia populated area corresponds to where Müller cells and their ensheathment of the Henle fibers is lost. Near the foveal center, 50 serial-sections, representing ~1.5 μm in Z-depth, and 336 serial-sections, representing ~10 μm in Z-depth along the MacTel zone border, were analyzed. Panels **(B, D)** are projections encompassing the full depth of each image volume showing the complete population of microglial cells identified.

**Figure 12:**
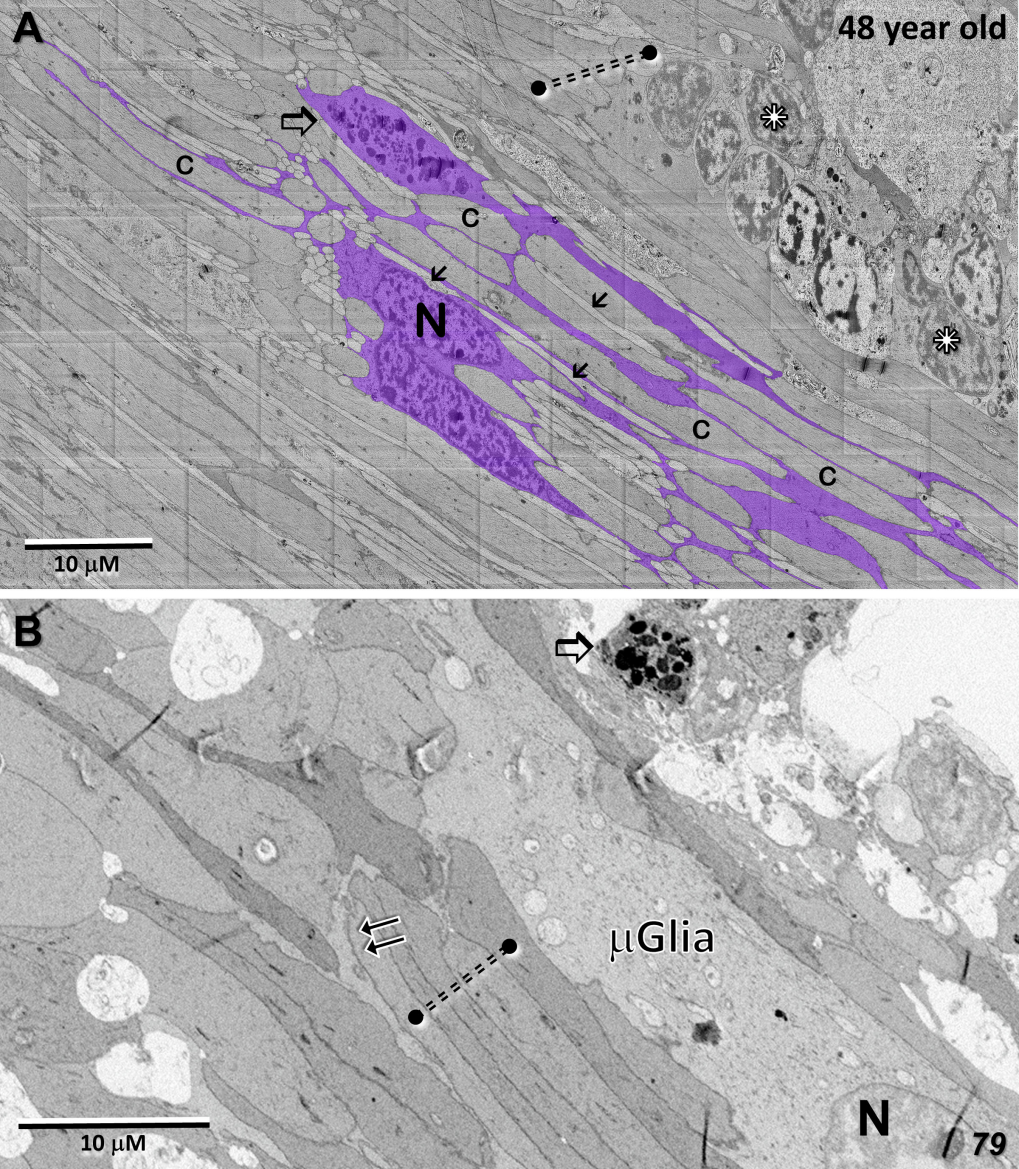
Microglial cell ensheathment of Henle fibers in the MacTel zone in both donor retinas. **(A)** A partially segmented microglial cell in the transition zone of the 48-year-old retina (purple). The multilobed shape of the nucleus (N) is characteristic of microglial cells. Continuous with the cell body, is an extensive network of fine processes that ensheathe the darker and thicker cone Henle fibers (c), as well as the thinner lighter Henle fibers of rods (arrows). A cytoplasmic swelling belonging to the microglial cell is filled with cellular debris and degradative structures (open arrow). Several cone Henle fibers are seen directly juxtaposed with each other, lacking any intervening glial separation normally provided by Müller cells (dashed double-line), along with a cluster of ectopic photoreceptor cell bodies (stars). **(B)** Like in the 48-year-old, microglial cells are found within the Henle fiber layer of the 79-year-old retina. Microglial processes surround some Henle fibers (double-arrows), with many directly juxtaposed with each other lacking any form of ensheathment (dashed double-line). A cellular debris containing swelling is also noted (open arrow).

Whereas microglial cells are described typically as populating sparsely the inner and outer plexiform layers in the normal retina (32), we have found significant numbers of microglial cells within the Henle fiber layer inside the MacTel zone. We have examined two regions from our 48YO donor, one nasal to the lesion near the fovea (**Figures 11A, B**), as well as along the temporal border of the MacTel zone (**Figures 11C, D**). Based on their distinctive nuclear and cytoplasmic characteristics (33), we mapped their distribution within a defined volume extending from the outer to the inner nuclear layers. In both regions, numerous microglial cells were found nearly exclusively within the confines of the Henle fiber layer, with few found more proximally in the outer plexiform layer. Strikingly, the border of the MacTel zone exhibits a precipitous drop in the microglial cell density, despite a continued inclusion of the Henle fiber layer within our volume (**Figures 11C, D**). We have shown previously (22) that the processes of Müller cells faithfully ensheathe the rod and cone Henle fibers outside the MacTel zone, and that this ensheathment stops abruptly as one enters into the MacTel zone, which coincides with where the Müller cells have largely degenerated. We have found that the processes of microglial cells within the Henle fiber layer also ensheathe the rod and cone axons. Expansions of these microglial processes filled with cellular debris are also often found, suggesting that along with the phagocytic and neuro-immune functions typically attributed to microglial cells, they may also play a structural and/or functional compensatory role normally performed by the Müller glial cells (**Figure 12A**). As seen in the 48YO retina, Müller cell ensheathment of the Henle fibers inside the MacTel zone of our 79YO is also lost, with many Henle fibers directly juxtaposed with each other, along with some intervening microglial cell processes (**Figure 12B**).

**Figure 13:**
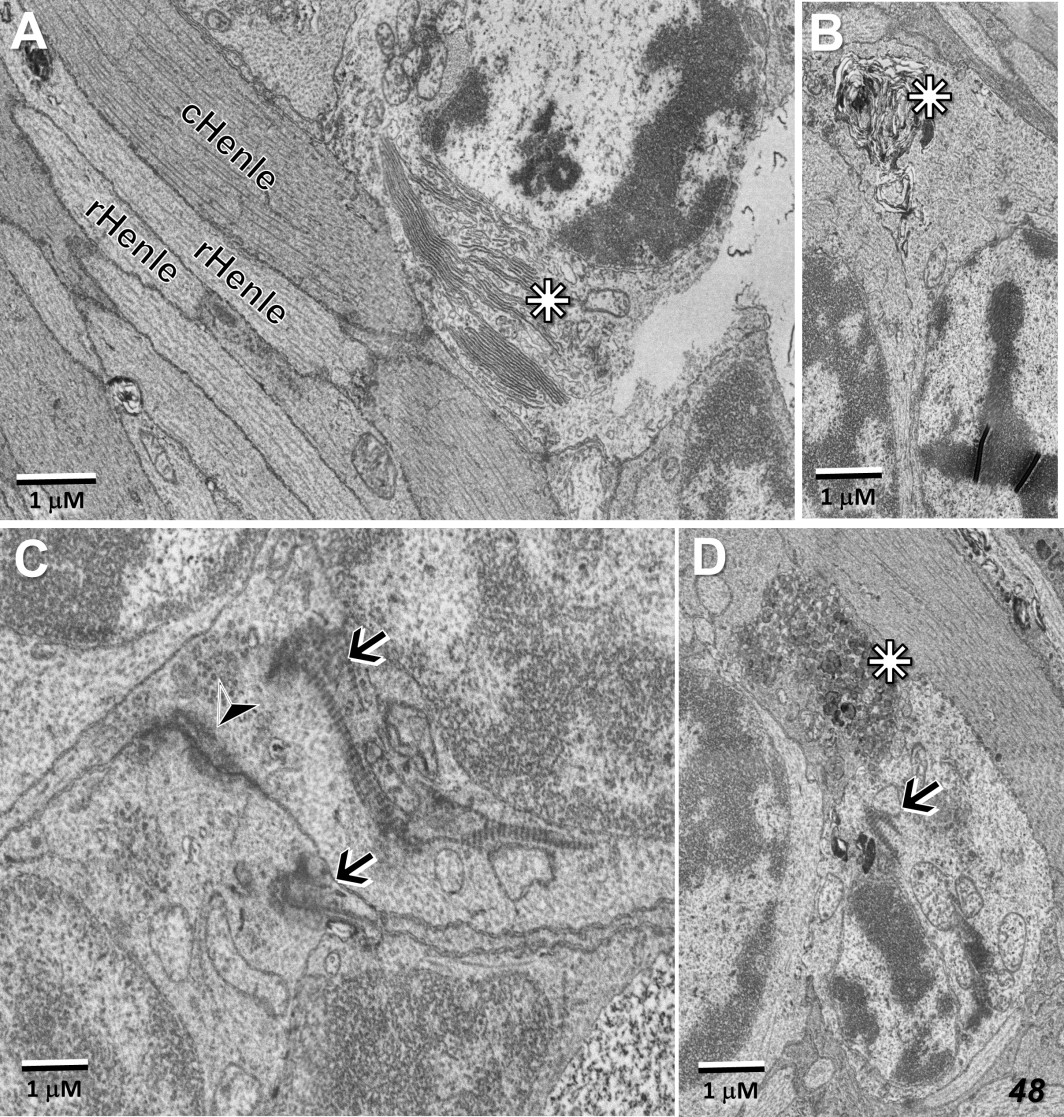
Ectopic photoreceptor cells in the Henle fiber layer along the border of the MacTel zone. Outer segment material near the cell nucleus (stars) may be well stacked **(A)**, distorted **(B)**, or vesicularized **(D)**. Basal-bodies and their associated striated rootlets (see also **Figure 5A**) are also typically found near the nuclei (arrows, **C, D**), as well as likely gap junctions between adjacent ectopic photoreceptor cells (arrow head, **C**). Rod and cone Henle fibers (r, cHenle) in direct juxtaposition with each other.

Also along the border of the MacTel zone, we described previously, clusters of photoreceptor cell bodies (some of which show pyknotic changes), cellular debris, and microglial cell processes, proximal to the outer nuclear layer within the Henle fiber layer. Common features of these displaced photoreceptor cell bodies are stacks of outer segment discs and degenerate outer segment material located ectopically adjacent to the cell nucleus (**Figures 13A, B, D**). Basal bodies, with their associated striated rootlets that would normally constitute the support structure of a connecting cilium (34 for review), and apparent gap junctions with juxtaposed photoreceptor cell bodies, are also common in these cell clusters, the latter perhaps representing a form of retinal remodeling (**Figures 13C, D**; 35, 36).

## Discussion

Our use of a multi-resolution targeted high-throughput connectomics approach has allowed us to survey the overall structural changes that occur across and beyond the macula region of two genetically related MacTel patients, with particular focus on mitochondrial integrity and glial-neuronal relationships.

Although the tissue preservation of the retina from the 79YO is not as good as that from the 48YO donor (likely a function of both age and other factors beyond our control), the main features of the MacTel disease process are consistent across both donors. Both retinas show specific mitochondrial changes throughout all retinal layers within and beyond the MacTel zone. Mitochondrial changes include those with condensed electron-dense structures interspersed with more normal looking cristae; mitochondria whose entire contents (cristae and matrix) are completely filled with electron-dense material, others are swollen with extensive empty matrix area and few cristae fragments, as well as those in various stages of degradation and mitophagy. Outside of the MacTel zone, most mitochondria showing some cristae loss appear to retain sufficient structural integrity to suggest maintained function. Interestingly, the outer mitochondrial membranes appear more resistant to degeneration then the inner mitochondrial membranes and their associated cristae. The outer membranes of mitochondria derive from the endoplasmic reticulum, versus the inner membranes which is where the principal enzymatic reactions occur in the organelle; perhaps a function of the systemic serine deficiency associated with MacTel. Within the MacTel zone, where the Müller glial cells are severely affected, ionic imbalances would also be expected. However, the electron-densities we observe differ in location and appearance from the calcium phosphate inclusions found in the mitochondrial matrix between cristae under conditions of ionic imbalance (37). There is evidence that mitochondrial and ER structure change with aging and neurodegenerative disease (38, 39), as does the interaction between these subcellular organelles. In aging optic nerve, mitochondria have been shown to be less numerous, but are longer and thicker. Such age-related changes in mitochondrial structure are qualitatively different from those we observe in MacTel eyes and our impression from study of both retinas is that the mitochondrial changes are the initial alterations in MacTel.

Oxidative stress and mitochondrial dysfunction are widely associated with diseases affecting the outer retina/RPE and elsewhere in the brain (40–44). Retraction of Müller cell processes in a retinal disease model has also been shown (45). Müller cells in the human macula, compared to those in the periphery, also appear to be more dependent upon serine for control of oxidative stress (21). Indeed, lowered levels of serine are associated with MacTel. Furthermore, reduced serine levels have been shown to correlate with increased levels of deoxysphingolipids (deoxySL), which are thought to be toxic to photoreceptors and lead to reduced visual function (46, 47).

In the lesion area, photoreceptors are lost (largely cones near the macular center). However, just beyond the lesion in both retinas, many are preserved. Nonetheless, we often do find significant pathology in the photoreceptor outer segments. In the MacTel retinas, compared to the well-ordered stacks seen in normal retinas, the outer segment discs become fragmented and extensively vesicularized, often showing discs distant from each other connected by membranous loops. Despite mitochondrial defects, the inner segments of both rods and cones are largely maintained, with intact basal bodies and connecting cilia, along with tight junctions with Müller cell processes (where present, constituting an outer limiting membrane) when outer segments are showing severe degeneration. Of significance, inner segments are known to be able to regenerate outer segments (48). This could explain adaptive optics evidence for the reappearance of cones near MacTel lesion areas over time (49).

Why do the inner segments remain relatively intact while the outer segments of the photoreceptors degenerate? One possibility explaining outer segment degeneration is that at low serine levels toxic deoxysphingolipids accumulate. This has been shown in mice on a low serine diet, which results in deoxysphingolipids accumulation, some photoreceptor cell death, and a reduction in the photopic ERG (46). Since serine is synthesized in both the RPE and Müller cells, the close proximity of the outer segments to the RPE might suggest that a loss of locally synthesized serine leads to outer segment degeneration (50).

Substantial increase in the risk of MacTel is associated with mutations in genes related to serine synthesis, and metabolomics studies indicate decreased serine levels in patients with MacTel. An increasing focus is on the dependence of retinal neurons on Müller cell based L-serine biosynthesis since retinal neurons (as do neurons throughout the CNS) are lacking in the rate limiting biosynthetic enzyme phosphoglycerate dehydrogenase/PHGDH (51–53). Shown specifically in Müller and other retinal cells, *de novo* L-serine synthesis is essential for sphingolipid metabolism as well as the maintenance of mitochondrial function (54, 55). It also provides the substrate for glycine and D-serine production, among other essential metabolic functions (56, 57). Not only has metabolomics analysis implicated defects in the L-serine metabolic pathway in the development of MacTel, but genome-wide association/GWAS studies have associated alterations in the PHGDH gene with early onset MacTel (58–60), with haploinsufficiency being causative in some patients (61). Furthermore, selective knock-down of PHGDH in mouse Müller cells results in reduced ERG responses along with photoreceptor degeneration (62).

Imaging the entirety of flatmount sections at intervals throughout the depth of each donor retina has allowed us to follow the MacTel lesions, defining their origin and vascular relationships. Likely following the loss of Müller cells in the MacTel zone and disruption of the outer limiting membrane, we observe a focal breakdown of Bruch’s membrane, infiltration of endothelial cells and other vascular elements from the choriocapillaris into the underlying pigment epithelial layer, along with an apparent “dragging” of pigment epithelial cells into the photoreceptor and other layers of the retina down to the ganglion cell layer. Typically, the vessels of the choriocapillaris are fenestrated, allowing relatively large molecules such as ferritin and horseradish peroxidase to pass through (63). However, we have shown previously that the cell membranes surrounding the lesion (likely endothelial cell) show extensive zonula adherens-like structures, suggesting that the blood-retinal barrier may be maintained proximal to Bruch’s membrane. Vascular elements from the deep, intermediate, and superficial retinal vascular plexes infiltrate and become a component of the lesion, as do pigment epithelial cells at all retinal depths. Although intraretinal cells derived from the RPE may dedifferentiate, losing some markers such as CRALBP and RPE65 (64), our direct observation of their origin and ultrastructural appearance supports the RPE origin of the pigmented cells throughout the retinal depth.

Although we were not able to visualize the extent of the MacTel zone in the 79YO donor eye based on the macular pigment profile (as we could for the 48YO), clinical evidence suggests that the size of the MacTel zone is relatively consistent and static across the patient population. In the 48YO, we have shown previously that there is a discrete border marking the extent of the MacTel zone. Just outside this border, Müller cell processes contribute to the outer limiting membrane, and comprehensively ensheathe the photoreceptor Henle fibers and synaptic terminals, as they do in normal retinas (25). Just inside the border, a modified outer limiting membrane consists largely of direct contacts between adjacent photoreceptor inner segments, and ensheathment of the Henle fibers and synaptic terminals is largely missing. This same pattern is seen in the 79YO retina, with a loss of Müller cell contribution to the outer limiting membrane occurring over a distance of less than 150 μm, along with a loss of Müller cell ensheathment of the Henle fibers. Strikingly, microglial cells, whose distal extent in the normal retina is limited to the outer plexiform layer (65), are found amongst the Henle fibers, often appearing to ensheathe them, as the lost Müller cell processes would have done. We were able to map the microglial cell population in the outer retina of the 48YO, finding that they are numerous, though restricted to the confines of the Henle fiber layer from near the foveal center to the edge of the MacTel zone, dropping precipitously at the border.

Based on ultrastructural features only resolvable by electron microscopy, and by partial segmentation of individual cells and their processes over relatively wide areas, we have identified a putative new role that microglial cells may be capable of playing in the retina – that being to provide the ensheathment of Henle fibers following the local loss of Müller cells, in addition to the phagocytic activity by Müller cells shown to occur in retinal disease models (66). Individual microglial cells that potentially provide an extensive network of Henle fiber ensheathing processes, are also actively involved in phagocytosis of cellular debris within discrete compartments or swellings along their processes, one of the roles more typically attributed to microglia. Along with supporting neuronal activity, synaptic function and wiring, control of neuroinflammation, among other roles, retinal microglia have also recently been shown to play a central role in the development of subretinal neovascularization (67, 68).

In summary, the methods and approach we describe in the present study have allowed us to provide insight into the progression and subcellular locus of a retinal neurodegenerative disease, namely MacTel. A similar approach could be applied to gain a deep understanding of other neurodegenerative diseases of the retina and elsewhere in the brain.

Along with subcellular changes, such as mitochondrial structure across and beyond the retinal extent with clinically relevant changes, we have shown significant changes in the interrelationships between retinal neurons and their glial partners. Numerous studies suggest that such interactions are important to understanding a range of retinal degenerative diseases including glaucoma, diabetic retinopathy, and age-related macular degeneration (AMD). Although various clinical imaging modalities have advanced significantly, multi-scale targeted high-throughput ultrastructural study provides a unique ability to probe the initial causes, cellular responses to, and progression of retinal degenerative diseases. Furthermore, determining the efficacy and precision of treatment modalities should also be amenable to this type of investigation.

## Data availability statement

The raw data supporting the conclusions of this article will be made available by the authors, without undue reservation.

## Ethics statement

Ethical approval was not required for the studies involving humans because fixed donor eyes were provided to us without any identifiers associated with the donors. The studies were conducted in accordance with the local legislation and institutional requirements. The human samples used in this study were acquired from donor eyes, were provided as fixed samples post-mortem. The donors consented to the use of their eyes for research purposes. Written informed consent to participate in this study was not required from the participants or the participants’ legal guardians/next of kin in accordance with the national legislation and the institutional requirements.

## Author contributions

CZ: Conceptualization, Data curation, Formal analysis, Funding acquisition, Investigation, Methodology, Project administration, Resources, Software, Supervision, Validation, Visualization, Writing – original draft, Writing – review & editing. PB: Funding acquisition, Resources, Writing – review & editing. RS: Data curation, Investigation, Methodology, Software, Visualization, Writing – review & editing. JL: Data curation, Funding acquisition, Methodology, Project administration, Resources, Software, Visualization, Writing – review & editing. JD: Conceptualization, Data curation, Formal analysis, Funding acquisition, Investigation, Methodology, Project administration, Resources, Software, Supervision, Validation, Visualization, Writing – original draft, Writing – review & editing.

## References

[1] Zhou J Chen B. Retinal cell damage in diabetic retinopathy. Cells. (2023) 12:1342. doi: 10.3390/cells12091342 37174742 PMC10177610

[2] Fletcher EL Phipps JA Ward MM Puthussery T Wilkinson-Berka JL. Neuronal and glial cell abnormality as predictors of progression of diabetic retinopathy. Curr Pharm Des. (2007) 13:2699–712. doi: 10.2174/138161207781662920 17897014

[3] Barber AJ Gardner TW Abcouwer SF. The significance of vascular and neural apoptosis to the pathology of diabetic retinopathy. Invest Ophthalmol Vis Sci. (2011) 52:1156–63. doi: 10.1167/iovs.10-6293 PMC3053099 21357409

[4] Tapia JC Kasthuri N Hayworth KJ Schalek R Lichtman JW Smith SJ. High-contrast en bloc staining of neuronal tissue for field emission scanning electron microscopy. Nat Protoc. (2012) 7:193–206. doi: 10.1038/nprot.2011.439 22240582 PMC3701260

[5] Hayworth KJ Morgan JL Schalek R Berger DR Hildebrand DG Lichtman JW. Imaging ATUM ultrathin section libraries with WaferMapper: a multi-scale approach to EM reconstruction of neural circuits. Front Neural Circuits. (2014) 8:68. doi: 10.3389/fncir.2014.00068 25018701 PMC4073626

[6] Kasthuri N Hayworth KJ Berger DR Schalek RL Conchello JA Knowles-Barley S. Saturated reconstruction of a volume of neocortex. Cell. (2015) 162:648–61. doi: 10.1016/j.cell.2015.06.054 26232230

[7] Pfeiffer RL Anderson JR Emrich DP Dahal J Sigulinsky CL Morrison HAB. Pathoconnectome analysis of Müller cells in early retinal remodeling. Adv Exp Med Biol. (2019) 1185:365–70. doi: 10.1007/978-3-030-27378-1_60 PMC7046339 31884639

[8] Pfeiffer RL Jones BW. Retinal pathoconnectomics: A window into neurodegeneration. Adv Exp Med Biol. (2023) 1415:297–301. doi: 10.1007/978-3-031-27681-1_43 37440048 PMC11342915

[9] Powner MB Gillies MC Tretiach M Scott A Guymer RH Hageman GS. Perifoveal müller cell depletion in a case of macular telangiectasia type 2. Ophthalmology. (2010) 117:2407–16. doi: 10.1016/j.ophtha.2010.04.001 PMC2974049 20678804

[10] Powner MB Gillies MC Zhu M Vevis K Hunyor AP Fruttiger M. Loss of Müller’s cells and photoreceptors in macular telangiectasia type 2. Ophthalmology. (2013) 120:2344–52. doi: 10.1016/j.ophtha.2013.04.013 23769334

[11] Cherepanoff S Killingsworth MC Zhu M Nolan T Hunyor AP Young SH. Ultrastructural and clinical evidence of subretinal debris accumulation in type 2 macular telangiectasia. Br J Ophthalmol. (2012) 96:1404–9. doi: 10.1136/bjophthalmol-2011-301009 PMC3512427 22976584

[12] Heeren TF Clemons T Scholl HP Bird AC Holz FG Charbel Issa P. Progression of vision loss in macular telangiectasia type 2. Invest Ophthalmol Vis Sci. (2015) 56:3905–12. doi: 10.1167/iovs.15-16915 26070062

[13] Okada M Robson AG Egan CA Sallo FB Esposti SD Heeren TFC. Electrophysiological characterization of macular telangiectasia type 2 and structure-function correlation. Retina. (2018) 38 Suppl 1:S33–42. doi: 10.1097/IAE.0000000000001746 28654458

[14] Gass JD Oyakawa RT. Idiopathic juxtafoveolar retinal telangiectasis. Arch Ophthalmol. (1982) 100:769–80. doi: 10.1001/archopht.1982.01030030773010 7082207

[15] Meleth AD Toy BC Nigam D Agrón E Murphy RP Chew EY. Prevalence and progression of pigment clumping associated with idiopathic macular telangiectasia type 2. Retina. (2013) 33:762–70. doi: 10.1097/IAE.0b013e3182695bb3 PMC3549320 23064429

[16] Leung I Sallo FB Bonelli R Clemons TE Pauleikhoff D Chew EY. Characteristics of pigmented lesions in type 2 idiopathic macular telangiectasia. Retina. (2018) 38 Suppl 1:S43–50. doi: 10.1097/IAE.0000000000001842 PMC5726940 29095354

[17] Spaide RF Marco RD Yannuzzi LA. Vascular distortion and dragging related to apparent tissue contraction in macular telangiectasis type 2. Retina. (2018) 38 Suppl 1:S51–60. doi: 10.1097/IAE.0000000000001694 28492432

[18] Sallo FB Leung I Zeimer M Clemons TE Dubis AM Fruttiger M. Abnormal retinal reflectivity to short-wavelength light in type 2 idiopathic macular telangiectasia. Retina. (2018) 38 Suppl 1:S79–88. doi: 10.1097/IAE.0000000000001728 PMC5726908 28644304

[19] Gass JD. Müller cell cone, an overlooked part of the anatomy of the fovea centralis: hypotheses concerning its role in the pathogenesis of macular hole and foveomacualr retinoschisis. Arch Ophthalmol. (1999) 117:821–3. doi: 10.1001/archopht.117.6.821 10369597

[20] Charbel Issa P Gillies MC Chew EY Bird AC Heeren TF Peto T. Macular telangiectasia type 2. Prog Retin Eye Res. (2013) 34:49–77. doi: 10.1016/j.preteyeres.2012.11.002 23219692 PMC3638089

[21] Zhang T Zhu L Madigan MC Liu W Shen W Cherepanoff S. Human macular Müller cells rely more on serine biosynthesis to combat oxidative stress than those from the periphery. Elife. (2019) 8:e43598. doi: 10.7554/eLife.43598 31036157 PMC6533082

[22] Zucker CL Bernstein PS Schalek RL Lichtman JW Dowling JE. A connectomics approach to understanding a retinal disease. Proc Natl Acad Sci U S A. (2020) 117:18780–7. doi: 10.1073/pnas.2011532117 PMC7414052 32699144

[23] Li B Gorusupudi A Arunkumar R Bernstein PS. Extraction, detection, and imaging of the macular carotenoids. Methods Enzymol. (2022) 674:185–213. doi: 10.1016/bs.mie.2022.05.001 36008007

[24] Zucker CL Dowling JE. Analyzing neural degeneration of the retina with connectomics. Neural Regener Res. (2022) 17:113–4. doi: 10.4103/1673-5374.314307 PMC8451567 34100445

[25] Burris C Klug K Ngo IT Sterling P Schein S. How Müller glial cells in macaque fovea coat and isolate the synaptic terminals of cone photoreceptors. J Comp Neurol. (2002) 453:100–11. doi: 10.1002/cne.10397 12357435

[26] Baena V Schalek RL Lichtman JW Terasaki M. Serial-section electron microscopy using automated tape-collecting ultramicrotome (ATUM). Methods Cell Biol. (2019) 152:41–67. doi: 10.1016/bs.mcb.2019.04.004 31326026 PMC8739344

[27] Berger DR Seung HS Lichtman JW. VAST (Volume annotation and segmentation tool): efficient manual and semi-automatic labeling of large 3D image stacks. Front Neural Circuits. (2018) 12:88. doi: 10.3389/fncir.2018.00088 30386216 PMC6198149

[28] Powner MB Woods SM Zhu M Gillies MC Bernstein PS Hageman GS. Fundus-wide subretinal and pigment epithelial abnormalities in macular telangiectasia type 2. Retina. (2018) 38 Suppl 1:S105–13. doi: 10.1097/IAE.0000000000001860 29045321

[29] Hori S Mukai N. Ultrastructural lesions of retinal pericapillary Müller cells in streptozotocin-induced diabetic rats. Albrecht Von Graefes Arch Klin Exp Ophthalmol. (1980) 213:1–9. doi: 10.1007/BF02391205 6906140

[30] Bianchi E Ripandelli G Taurone S Feher J Plateroti R Kovacs I. Age and diabetes related changes of the retinal capillaries: An ultrastructural and immunohistochemical study. Int J Immunopathol Pharmacol. (2016) 29:40–53. doi: 10.1177/0394632015615592 26604209 PMC5806738

[31] Fehér J Taurone S Spoletini M Biró Z Varsányi B Scuderi G. Ultrastructure of neurovascular changes in human diabetic retinopathy. Int J Immunopathol Pharmacol. (2018) 31:394632017748841. doi: 10.1177/0394632017748841 29251013 PMC5849217

[32] Boycott BB Hopkins JM. Microglia in the retina of monkey and other mammals: its distinction from other types of glia and horizontal cells. Neuroscience. (1981) 6:679–88. doi: 10.1016/0306-4522(81)90151-2 6165924

[33] Goyal M Bordt AS Neitz J Marshak DW. Trogocytosis of neurons and glial cells by microglia in a healthy adult macaque retina. Sci Rep. (2023) 13:633. doi: 10.1038/s41598-023-27453-2 36635325 PMC9837165

[34] Wensel TG Potter VL Moye A Zhang Z Robichaux MA. Structure and dynamics of photoreceptor sensory cilia. Pflugers Arch. (2021) 473:1517–37. doi: 10.1007/s00424-021-02564-9 PMC11216635 34050409

[35] Pfeiffer RL Anderson JR Dahal J Garcia JC Yang JH Sigulinsky CL. A pathoconnectome of early neurodegeneration: Network changes in retinal degeneration. Exp Eye Res. (2020) 199:108196. doi: 10.1016/j.exer.2020.108196 32810483 PMC7554222

[36] Leinonen HO Bull E Fu Z. Neural and Müller glial adaptation of the retina to photoreceptor degeneration. Neural Regener Res. (2023) 18:701–7. doi: 10.4103/1673-5374.354511 PMC9700092 36204825

[37] Greenawalt JW Rossi CS Lehninger AL. Effect of active accumulation of calcium and phosphate ions on the structure of rat liver mitochondria. J Cell Biol. (1964) 23:21–38. doi: 10.1083/jcb.23.1.21 14228516 PMC2106507

[38] Hara Y Yuk F Puri R Janssen WG Rapp PR Morrison JH. Estrogen restores multisynaptic boutons in the dorsolateral prefrontal cortex while promoting working memory in aged rhesus monkeys. J Neurosci. (2016) 36:901–10. doi: 10.1523/JNEUROSCI.3480-13.2016 PMC4719022 26791219

[39] Zhang L Trushin S Christensen TA Bachmeier BV Gateno B Schroeder A. Altered brain energetics induces mitochondrial fission arrest in Alzheimer’s Disease. Sci Rep. (2016) 6:18725. doi: 10.1038/srep18725 26729583 PMC4700525

[40] Fritsche LG Loenhardt T Janssen A Fisher SA Rivera A Keilhauer CN. Age-related macular degeneration is associated with an unstable ARMS2 (LOC387715) mRNA. Nat Genet. (2008) 40:892–6. doi: 10.1038/ng.170 18511946

[41] Golestaneh N Chu Y Cheng SK Cao H Poliakov E Berinstein DM. Repressed SIRT1/PGC-1α pathway and mitochondrial disintegration in iPSC-derived RPE disease model of age-related macular degeneration. J Transl Med. (2016) 14:344. doi: 10.1186/s12967-016-1101-8 27998274 PMC5175395

[42] Stahon KE Bastian C Griffith S Kidd GJ Brunet S Baltan S. Age-related changes in axonal and mitochondrial ultrastructure and function in white matter. J Neurosci. (2016) 36:9990–10001. doi: 10.1523/JNEUROSCI.1316-16.2016 27683897 PMC5039264

[43] Lefevere E Toft-Kehler AK Vohra R Kolko M Moons L Van Hove I. Mitochondrial dysfunction underlying outer retinal diseases. Mitochondrion. (2017) 36:66–76. doi: 10.1016/j.mito.2017.03.006 28365408

[44] Ferrington DA Fisher CR Kowluru RA. Mitochondrial defects drive degenerative retinal diseases. Trends Mol Med. (2020) 26:105–18. doi: 10.1016/j.molmed.2019.10.008 PMC6938541 31771932

[45] Allingham MJ Cousins SW Mettu PS. Mitochondrial dysfunction causes retraction of Müller cell lateral processes. Invest Ophthalmol Vis Sci. (2021) 62:1685.

[46] Gantner ML Eade K Wallace M Handzlik MK Fallon R Trombley J. Serine and lipid metabolism in macular disease and peripheral neuropathy. N Engl J Med. (2019) 381:1422–33. doi: 10.1056/NEJMoa1815111 PMC7685488 31509666

[47] Bonelli R Woods SM Ansell BRE Heeren TFC Egan CA Khan KN. Systemic lipid dysregulation is a risk factor for macular neurodegenerative disease. Sci Rep. (2020) 10:12165. doi: 10.1038/s41598-020-69164-y 32699277 PMC7376024

[48] Dowling JE Wald G. The biological function of vitamin a acid. Proc Natl Acad Sci U S A. (1960) 46:587–608. doi: 10.1073/pnas.46.5.587 16590647 PMC222881

[49] Wang Q Tuten WS Lujan BJ Holland J Bernstein PS Schwartz SD. Adaptive optics microperimetry and OCT images show preserved function and recovery of cone visibility in macular telangiectasia type 2 retinal lesions. Invest Ophthalmol Vis Sci. (2015) 56:778–86. doi: 10.1167/iovs.14-15576 PMC4315435 25587056

[50] Sinha T Ikelle L Naash MI Al-Ubaidi MR. The intersection of serine metabolism and cellular dysfunction in retinal degeneration. Cells. (2020) 9:674. doi: 10.3390/cells9030674 32164325 PMC7140600

[51] Yamasaki M Yamada K Furuya S Mitoma J Hirabayashi Y Watanabe M. 3-Phosphoglycerate dehydrogenase, a key enzyme for l-serine biosynthesis, is preferentially expressed in the radial glia/astrocyte lineage and olfactory ensheathing glia in the mouse brain. J Neurosci. (2001) 21:7691–704. doi: 10.1523/JNEUROSCI.21-19-07691.2001 PMC6762884 11567059

[52] Furuya S. An essential role for *de novo* biosynthesis of L-serine in CNS development. Asia Pac J Clin Nutr. (2008) 17 Suppl 1:312–5. 18296366

[53] Ehmsen JT Ma TM Sason H Rosenberg D Ogo T Furuya S. D-serine in glia and neurons derives from 3-phosphoglycerate dehydrogenase. J Neurosci. (2013) 33:12464–9. doi: 10.1523/JNEUROSCI.4914-12.2013 PMC3721849 23884950

[54] Lucas S Chen G Aras S Wang J. Serine catabolism is essential to maintain mitochondrial respiration in mammalian cells. Life Sci Alliance. (2018) 1:e201800036. doi: 10.26508/lsa.201800036 30456347 PMC6238390

[55] Zhang T Gillies MC Madigan MC Shen W Du J Grünert U. Disruption of *de novo* serine synthesis in Müller cells induced mitochondrial dysfunction and aggravated oxidative damage. Mol Neurobiol. (2018) 55:7025–37. doi: 10.1007/s12035-017-0840-8 29383682

[56] Hirabayashi Y Furuya S. Roles of L-serine and sphingolipid synthesis in brain development and neuronal survival. Prog Lipid Res. (2008) 47:188–203. doi: 10.1016/j.plipres.2008.01.003 18319065

[57] Gao X Lee K Reid MA Sanderson SM Qiu C Li S. Serine availability influences mitochondrial dynamics and function through lipid metabolism. Cell Rep. (2018) 22:3507–20. doi: 10.1016/j.celrep.2018.03.017 PMC6054483 29590619

[58] Scerri TS Quaglieri A Cai C Zernant J Matsunami N Baird L. Genome-wide analyses identify common variants associated with macular telangiectasia type 2. Nat Genet. (2017) 49:559–67. doi: 10.1038/ng.3799 28250457

[59] Bonelli R Jackson VE Prasad A Munro JE Farashi S Heeren TFC. Identification of genetic factors influencing metabolic dysregulation and retinal support for MacTel, a retinal disorder. Commun Biol. (2021) 4:274. doi: 10.1038/s42003-021-01788-w 33654266 PMC7925591

[60] Bonelli R Ansell BRE Lotta L Scerri T Clemons TE Leung I. Genetic disruption of serine biosynthesis is a key driver of macular telangiectasia type 2 aetiology and progression. Genome Med. (2021) 13:39. doi: 10.1186/s13073-021-00848-4 33750426 PMC7945323

[61] Eade K Gantner ML Hostyk JA Nagasaki T Giles S Fallon R. Serine biosynthesis defect due to haploinsufficiency of PHGDH causes retinal disease. Nat Metab. (2021) 3:366–77. doi: 10.1038/s42255-021-00361-3 PMC8084205 33758422

[62] Shen W Lee SR Mathai AE Zhang R Du J Yam MX. Effect of selectively knocking down key metabolic genes in Müller glia on photoreceptor health. Glia. (2021) 69:1966–86. doi: 10.1002/glia.24005 33835598

[63] Bernstein MH Hollenberg MJ. Fine structure of the choriocappillaris and retinal capillaries. Invest Ophthalmol. (1965) 4:1016–25. 5321525

[64] Yasvoina M Yang Q Woods SM Heeren T Comer GM A Egan C. Intraretinal pigmented cells in retinal degenerative disease. Br J Ophthalmol. (2023) 107:1736–43. doi: 10.1136/bjophthalmol-2021-320392 35301216

[65] Singaravelu J Zhao L Fariss RN Nork TM Wong WT. Microglia in the primate macula: specializations in microglial distribution and morphology with retinal position and with aging. Brain Struct Funct. (2017) 222:2759–71. doi: 10.1007/s00429-017-1370-x PMC5542874 28213784

[66] Sakami S Imanishi Y Palczewski K. Müller glia phagocytose dead photoreceptor cells in a mouse model of retinal degenerative disease. FASEB J. (2019) 33:3680–92. doi: 10.1096/fj.201801662R PMC6404573 30462532

[67] Savage JC Picard K González-Ibáñez F Tremblay MÈ. A brief history of microglial ultrastructure: distinctive features, phenotypes, and functions discovered over the past 60 Years by electron microscopy. Front Immunol. (2018) 9:803. doi: 10.3389/fimmu.2018.00803 29922276 PMC5996933

[68] Usui-Ouchi A Usui Y Kurihara T Aguilar E Dorrell MI Ideguchi Y. Retinal microglia are critical for subretinal neovascular formation. JCI Insight. (2020) 5:e137317. doi: 10.1172/jci.insight.137317 32437334 PMC7406258

