# Supplementary figures and images for: High-throughput ultrastructural analysis of macular telangiectasia type 2
Source: Front Ophthalmol (Lausanne). 2024 Jul 30;4:1428777. doi: 10.3389/fopht.2024.1428777 (PMC11319912; doi:10.3389/fopht.2024.1428777)

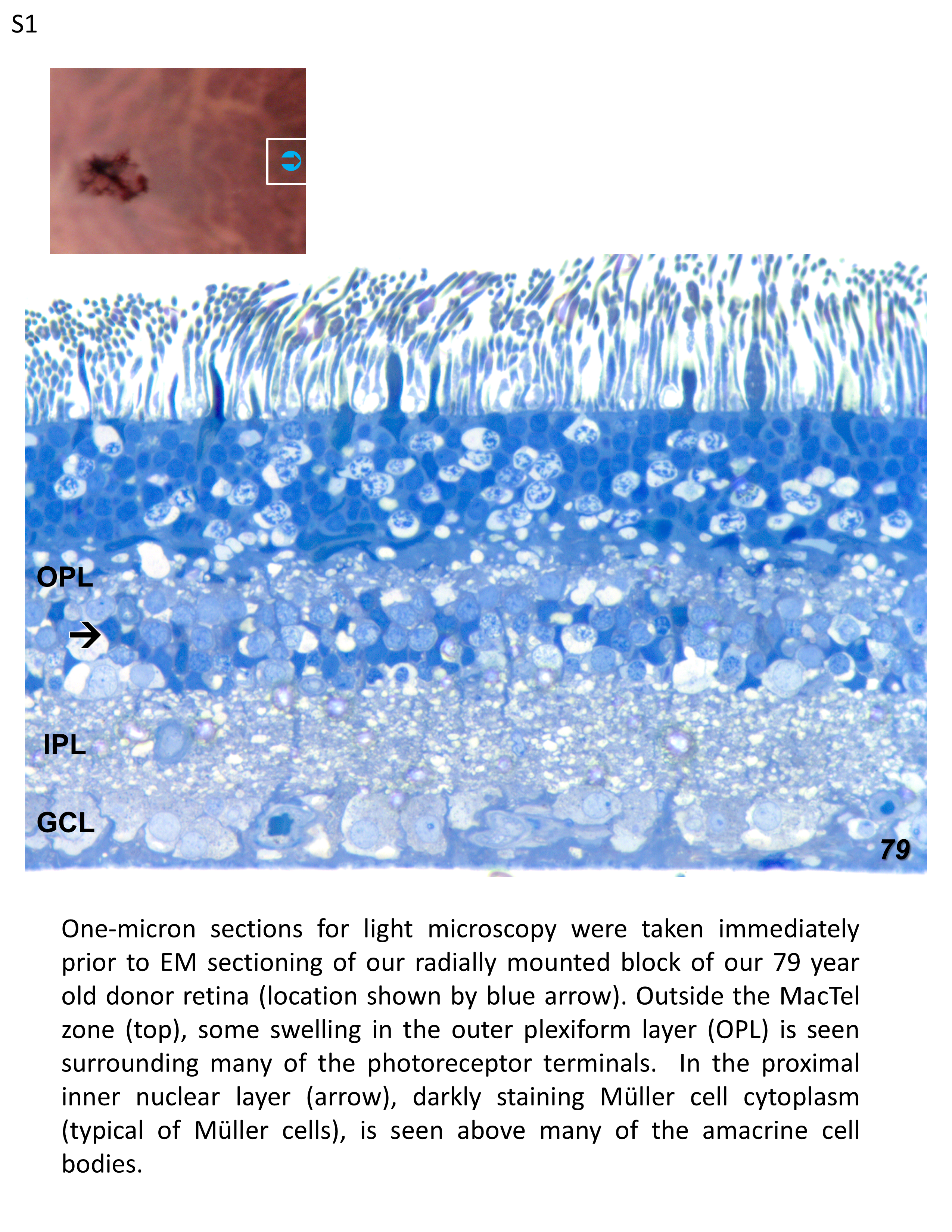

Supplement: Supplementary file 1 [file Image_1.tif]

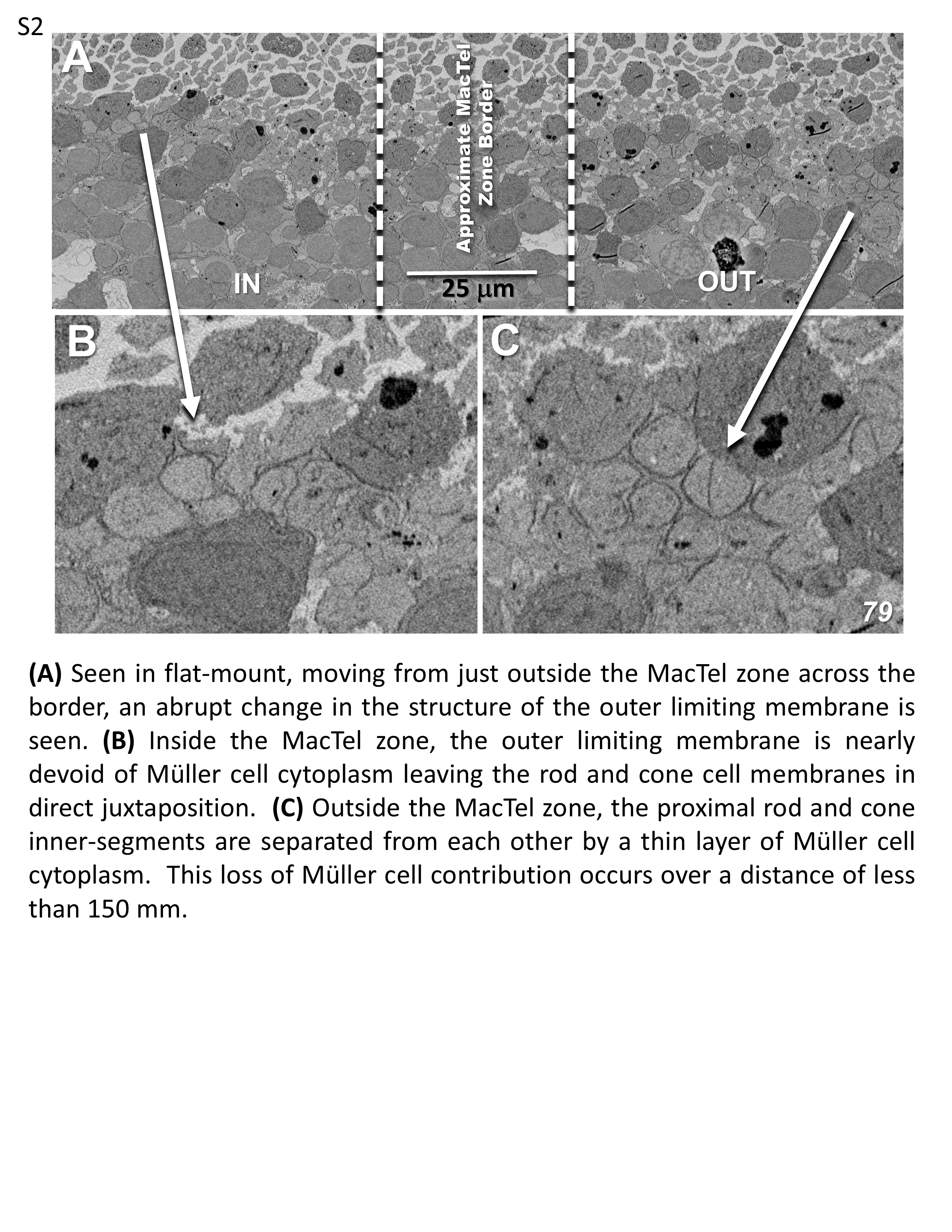

Supplement: Supplementary file 2 [file Image_2.tif]

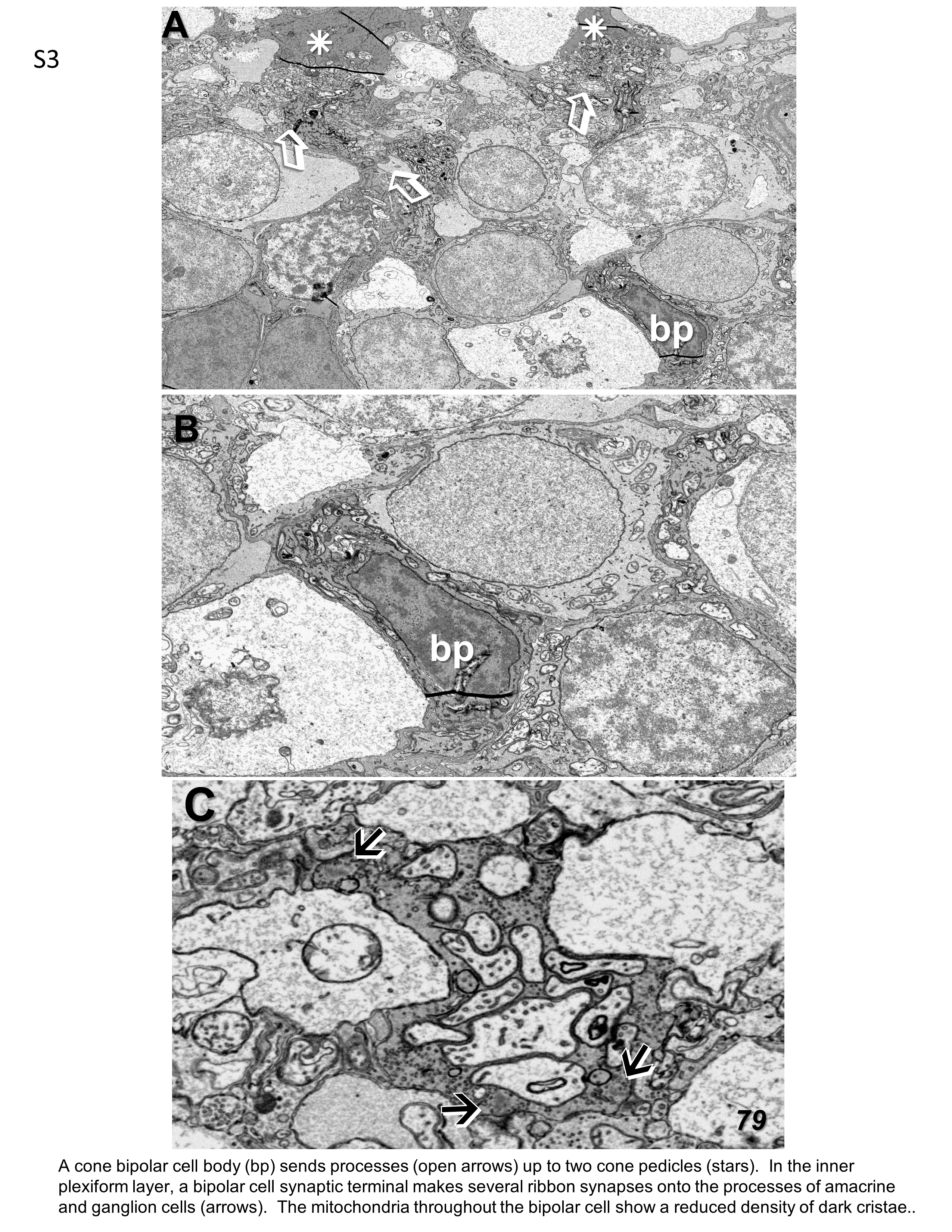

Supplement: Supplementary file 3 [file Image_3.tif]
